# Polyphasic characterization of a novel hot-spring cyanobacterium *Thermocoleostomius sinensis* gen et sp. nov. and genomic insights into its carbon concentration mechanism

**DOI:** 10.3389/fmicb.2023.1176500

**Published:** 2023-07-26

**Authors:** Ying Jiang, Jie Tang, Xiangjian Liu, Maurycy Daroch

**Affiliations:** ^1^School of Environment and Energy, Peking University Shenzhen Graduate School, Shenzhen, China; ^2^School of Food and Bioengineering, Chengdu University, Chengdu, Sichuan, China

**Keywords:** *Thermocoleostomius*, 16S rRNA, 16S-23 ITS, CO_2_-concentrating mechanism, *Thermophilic cyanobacterium*, *Oculatellaceae*, *Leptolyngbyaceae*, *Albertania*

## Abstract

Thermophilic cyanobacteria play a crucial role as primary producers in hot spring ecosystems, yet their microbiological, taxonomic, and ecological characteristics are not extensively studied. This study aimed to characterize a novel strain of thermophilic cyanobacteria, PKUAC-SCTA174 (A174), using a combination of traditional polyphasic methods and modern genomic-based approaches. The study included 16S rRNA-based phylogeny, ITS secondary structure prediction, morphological and habitat analyses, as well as high-quality genome sequencing with corresponding phylogenomic analyses. The results of the 16S rRNA, 16S-23S ITS secondary structure, morphology, and habitat analyses supported the classification of the strain as a member of a novel genus within the family *Oculatellaceae*, closely related to *Albertania* and *Trichotorquatus*. Genomic analysis revealed the presence of a sophisticated carbon-concentrating mechanism (CCM) in the strain, involving two CO_2_ uptake systems NDH-I_3_, and NDH-I_4_, three types of bicarbonate transporters (BCT1, bicA, sbtA,) and two distinct putative carboxysomal carbonic anhydrases (*ccaA1* and *ccaA2*). The expression of CCM genes was investigated with a CO_2_ shift experiment, indicating varying transcript abundance among different carbon uptake systems. Based on the comprehensive characterization, the strain was delineated as *Thermocoleostomius sinensis*, based on the botanical code. The study of the complete genome of strain A174 contributes valuable insights into the genetic characteristics of the genus *Thermocoleostomius* and related organisms and provides a systematic understanding of thermophilic cyanobacteria. The findings presented here offer valuable data that can be utilized for future research in taxogenomics, ecogenomics, and geogenomics.

## Introduction

Thermophilic cyanobacteria are essential primary producers of geothermal ecosystems that profoundly impact the ecology and biological productivity of these sites (Esteves-Ferreira et al., 2018). In recent years, there has been increased interest in these extremophilic organisms with a focus on their ecology, taxonomy, genomics, and biotechnology as donors of useful enzymes and metabolites (Liang et al., 2018; Patel et al., 2019) and microbial cell factories for carbon photo valorization (Liang et al., 2019). Despite an observable increase in interest in this group of photosynthetic extremophiles, many organisms remain relatively poorly described compared to their mesophilic counterparts. In recent years, low costs and increased availability of next-generation sequencing has led to increases in metagenomic studies of hot spring microbial communities (Alcorta et al., 2020). Numerous Metagenome-Assembled Genomes (MAGs) of thermophilic cyanobacteria have been recovered from these sequencing projects highlighting genomic features of new thermophilic microorganisms and potential mechanisms of adaptations to the environment (Chen et al., 2020; Kees et al., 2022). Until recently, it was difficult to conduct fundamental and applied studies on thermophilic strains due to the lack of well-described thermophilic isolates that contain complete genomic, taxonomic, physiological, and morphological data. However, as more studies characterizing thermophilic strains have been published, the new genera have been proposed (Perona et al., 2022). Simultaneously, thermophilic cyanobacteria share many of the same challenges regarding their taxonomy to their mesophilic counterparts, most notably simple morphology (Komárek and Anagnostidis, 2005). Those features, often difficult to identify and plastic, like colony and filament morphology, types and sizes of cells, and characteristic features of sheaths, aerotopes, or branches make their assignment particularly challenging. The absence of sufficient genetic, physiological, or morphological data exacerbates this problem. To effectively identify cyanobacterial isolates and resolve their precise taxonomic status, a polyphasic approach combining multiple datasets is necessary (Raabova et al., 2019; Shalygin et al., 2020).

In the past few years, there has been a significant increase in the discovery of new species of cyanobacteria (Casamatta, 2023; Strunecký et al., 2023). This can be also observed among thermophilic strains where simple unicellular organisms such as *Thermosynechococcus, Synechococcus*, and *Thermostichus* (Komárek et al., 2020; Tang et al., 2022c) and filamentous strains exhibiting *Leptolyngbya*-like morphology underwent a series of revisions in recent years (Sciuto and Moro, 2016; Mai et al., 2018). Meanwhile, extensive revisions have also been made in the family *Oculatellaceae*, and numerous clades and new genera have been delineated from the strains previously assigned to *Leptolyngbya*, e.g., *Drouetiella* (Mai et al., 2018), *Thermoleptolyngbya* (Sciuto and Moro, 2016), *Timaviella* (Mai et al., 2018), and *Trichotorquatus* (Pietrasiak et al., 2021).

However, despite the wide and rapid deployment of next-generation sequencing technology (NGS) in almost all areas of microbiology, cyanobacterial taxonomy often lacks sufficient genomic components. This can lead to taxonomic revisions of a well-established cyanobacterial taxon based exclusively on single-gene sequencing and microscopic observations (Nowicka-Krawczyk et al., 2019). To enhance the credibility of taxonomic revisions, it is crucial, in our opinion, to incorporate comparative genomic analyses to strain descriptions that include morphological and ecological data. Examples of revisions that have been backed by genomic data include the delineation of *Thermoleptolynbya* (Sciuto and Moro, 2016) and *Trichothermofontia* (Tang et al., 2023). While many of the delineations that originally did not include comparative genomics were already confirmed and expanded thanks to those datasets (Strunecký et al., 2023), in many other instances it was not possible due to sequence unavailability. With the renewed interest in cyanobacterial taxonomy in recent years, it is important that those consolidated community efforts based on a polyphasic approach also include genomic and physiological data, to better guide taxonomic revisions in future.

In this study, an isolate PKUAC-SCTA174 (A174 thereafter), isolated from the Erdaoqiao Hot Spring in Ganzi Prefecture, western Sichuan Province of China, previously suggested as a potential new genus (Tang et al., 2018a) is described. The strain is the third potential novel organism from the hot spring after *Thermoleptolyngbya sichuanensis* (Tang et al., 2021) and *Leptodesmis sichuanensis* (Tang et al., 2022a) described earlier. A complete dataset containing genomic, morphological, and physiological data of the isolated thermophilic filamentous strain is presented. On the basis of the data and utilizing a polyphasic approach, a new genus is proposed *Thermocoleostomius sinensis* gen. et sp. nov., based on the botanical code. Finally, the genes responsible for the strain's sophisticated carbon concentration mechanism (CCM) were studied in response to changes in inorganic carbon availability.

## Materials and methods

### Origins, cultivation, deposition, and basic physiological assessment

The strain A174 was initially isolated from light green biofilm deposited on the surface of calcareous sinter in the Erdaoqiao Hot Spring in the Ganzi Prefecture, western Sichuan Province, China. Original sampling was performed on 12 May 2016 under environmental conditions including an ambient temperature of 15°C, a relative humidity of 71%, and a light intensity of ~1,000 lux. The temperature and pH of the thermal spring were 40.8°C and 6.32, respectively. The total dissolved solids in the spring were 447 mmol L^−1^. The hot spring sampling site description and preliminary taxonomic identification have been reported earlier earlier (Tang et al., 2018a,b). The uni-cyanobacterial culture was routinely cryopreserved as 10% DMSO in BG11 stocks at −80°C. Unless stated otherwise, the pre-cultures for the experiments were established as 150 ml BG-11 (Stanier et al., 1971) medium in 500 ml Erlenmeyer flasks agitated at 100 rpm in an illuminated shaking incubator (MGC-450BP-2, Yiheng, Shanghai, China) for 14 days. Unless stated otherwise, the light parameters were set at 45 μmol m^−2^ s^−1^ provided by cool white light fluorescent tubes, temperature 45°C, and photoperiod 12L: 12D. The strain was deposited in the Freshwater Algae Culture Collection at the Institute of Hydrobiology (FACHB-collection) with an accession number FACHB-3572. Simultaneously, the dried inactive holotype was deposited in the Herbarium of North Minzu University with the voucher number: NMU00174 along with other strains collected in Sichuan Ganzi Prefecture, which belonged to other genera and were described earlier and deposited in the same herbarium with voucher numbers: NMU00183 (A183 strain; Tang et al., 2021), NMU00121 (A121 strain; Tang et al., 2022a), NMU00231 (B231 strain), and NMU00412 (E412 strain; Tang et al., 2022b). All the herbarium specimens were prepared by harvesting and gently drying on the filter paper their biomass before deposition. Strain capacity to utilize various nitrogen and carbon sources was tested as follows: the strain was cultured in a series of concentrations of sodium bicarbonate (0, 0.1, 0.3, 0.5, 0.7, and 1 M) and nitrogen sources, including sodium nitrate (0, 0.075, 0.5, 1.5, 5, and 7.5 g L^−1^) and sodium nitrite (1.218 g L^−1^). Batch experiments were performed in 250 ml flasks and 50 ml breathable capped tubes containing 100 and 25 ml culture medium, respectively. The control groups were cultured in a standard BG11 medium. Similar sizes and numbers of cell pellets containing trichoid granules were inoculated in the experimental and control groups. The temperature was maintained at 45°C, and the experiment was performed continuously for 21 days, with adjustment for the evaporative losses.

### Genome sequencing, assembly, and annotation of strain A174

Genomic DNA was extracted as previously described (Tang et al., 2021) and tested for integrity using agarose gel electrophoresis. The whole-genome sequencing was conducted using a combination of long-read Oxford Nanopore Technologies (ONT) performed with the PromethION sequencer, and Illumina short-read PE150 approach using the NovaSeq 6000 sequencer. For ONT sequencing, the libraries were generated using SQK-LSK109 Kit according to the manufacturer's guidelines. ONT sequencing yielded 1,387,280,929 bp of an average read length of 10,405 bp. These long-reads were used to assemble the draft circular contig of the genome using Flye v2.7 (Kolmogorov et al., 2019) plugin to the commercially available Geneious Prime 2022.2 package (Kearse et al., 2012). Subsequently, the draft contig was error-corrected with 6,098,395 filtered PE150 reads (clean data) generated with short-read technology, using Geneious mapper on default settings. Finally, the final genome was annotated using a customized pipeline, as described before (Tang et al., 2019). In short, the gene prediction and annotation were automatically performed using the NCBI prokaryotic genome annotation pipeline (O'Leary et al., 2016) and polished utilizing the RAST annotation system (Brettin et al., 2015) to minimize poor calls. The genome annotation of strain A174 was summarized in Supplementary Table 1. The complete genome was deposited in GenBank under accession number CP113797.

### Phylogenetic reconstruction of 16S rRNA

The sequences of the 16S rRNA gene were extracted from the complete genome sequence of the A174 strain. The reference sequences with high similarity to the A174 were selected based on the BLAST algorithm from the GenBank database or by identifying with the genera in the family *Oculatellaceae*. Each sequence was trimmed to a similar length of ~1,100 bp, aligned with ClustalW, refined by adjustment of poorly aligned regions, and used to generate a phylogenetic tree representing the taxonomic assignment of the A174 strain. Meanwhile, the 16S rRNA gene sequence similarities between species were calculated based on pairwise alignment using an online calculator from enveomics collection toolbox (Rodriguez-R and Konstantinidis, 2016). Independent phylogenetic relationships of cyanobacteria species within the order *Synechococcales* (including family *Oculatellaceae* and *Leptolyngbyaceae*) were simultaneously conducted using PhyML V3.0 (Guindon et al., 2010) and Bayesian analysis. The nexus file generated from the alignment was run in MrBayes 3.2.7a (Ronquist et al., 2012), available on CIPRES Science Gateway (Miller et al., 2010). The best-fit substitution evolutionary model was chosen by jModelTest based on the Bayesian Information Criterion. Bayesian analysis was run using the TVM + invariant + gamma (TVM+I+R) substitution model with the parameters: NST = 6, Rates = invgamma, and MCMC Ngen = 10,000,000. An initial 25% of samples were discarded as the burn-in fraction. The potential scale reduction factor (PSRF) for parameter values was 1.00, which realized the purpose of convergent statistics. Maximum likelihood was conducted using PhyML, employing the GTR model, with 1,000 bootstrap replications. Bayesian inference results showed the best result to classify the family and the genus and were used to map correct phylogenetic distances and evaluate the relative support of branches. All phylogenies were visualized using FigTree (Rambaut, 2007) and subsequently annotated in Adobe Illustrator.

### Prediction of secondary structures

The 16S−23S intergenic spacer (ITS) region was extracted from the annotated genome of the strain and analyzed for the presence of conserved domains. The conserved D1–D1′ domain and variable domains V2 and boxB of the 16S-23S ITS region were identified as described before (Iteman et al., 2000), and their secondary structures were computed with RNAstructure and visualized with Structure Editor 1.0 (Mathews, 2014). The tRNA sequences were identified using tRNA scan-SE v.1.3.1 (Lowe and Eddy, 1997).

### Genome-based analyses

A series of pairwise genomic comparisons were performed to further investigate the taxonomic relationships between A174 and related strains. The nucleotide and amino acid sequences were retrieved from GenBank databases. Pairwise average nucleotide identity (ANI) and average amino acid identity (AAI) were calculated for the A174 genome against both closely related strains and focus taxa. ANI/AAI parameters were calculated using a publicly available algorithm provided through the Environmental Microbial Genomics Laboratory at Georgia Tech (http://enve-omics.ce.gatech.edu/index) and visualized as a matrix table. Further genome similarity was compared using digital DNA–DNA hybridization (dDDH) between pairs of genomes. The estimated DDH distances were calculated using an online tool (Meier-Kolthoff et al., 2022) based on a generalized linear model (GLM) by submitting and comparing two genomes using the BLAT program to obtain HSPs/MUMs (high-scoring segment pair/ maximal matches that are unique in both sequences) (Chatterjee et al., 2015) and infer distances using formula most suitable for genomes.

### Phylogenomic reconstruction

Concatenated protein sequences generated with the shared single-copy genes among all focus taxa were used to ascertain the phylogenomic position of strain A174 essentially as described before (Tang et al., 2022a). In short, the homologous gene clusters were identified with OrthoMCL (Li et al., 2003) and concatenated. The resultant multisequence alignment was generated using MAFFT v7453 (Katoh and Standley, 2013) and analyzed for phylogenomic inference using IQ-TREE v2.1.3 (Minh et al., 2020). Subsequently, 546 protein models were used to create an optimal substitution model using a ModelFinder module of IQ-TREE. The final assessment of the generated tree topology was performed with UltraFast Bootstrap (Thi Hoang et al., 2017), employing 1000 bootstrap tests (replicates).

### Microscopic analysis

The light micrographs of the A174 strain were obtained from ~5 μl of a healthy 2-week-old culture grown at standard conditions at 400 × magnification using light microscopy (LM, DP72, OLYMPUS, Japan) equipped with an image acquisition system (U-TV0.63XC, OLYMPUS, Japan). Scanning electron microscopy (SEM) and transmission electron microscopy (TEM) micrographs were generated using the protocols described earlier (Tang et al., 2021). In short, the cells for SEM were washed in phosphate-buffered saline (PBS), fixed for 2 h in fixation solution (Servicebio, G1102), post-fixed with 1% OsO_4_, and dehydrated with ethanol and isoamyl acetate before taking micrographs with Hitachi, Tokyo, Japan, SU8100. Cells for TEM were additionally embedded in agarose and cut to 60–80 nm thin layers with ultra-microtome Leica EM UC7 (Leica, Wetzlar, Germany), stained with 2% uranium acetate saturated alcohol solution and lead citrate for 8 min, and examined using TEM (Hitachi, HT7800).

### Investigation of the carbon concentration mechanism in strain A174

The carbon concentration mechanism (CCM) components were identified as previously described (Tang et al., 2022c). In short, the 28 well-described protein sequences involved in the CCM of a model strain *Synechocystis* sp. PCC 6803 were retrieved as a reference. The orthologous gene set of the A174 strain was obtained using bidirectional best hit methodology employing *E*-value cutoff of 1E-6, ≥30% identity, and 70% coverage with BLASTP and subsequently manually curated for accuracy using annotated genomes and summarized in Supplementary Table 2. Nucleotide sequences of Rubisco large subunit (*rbcL*), carboxysome shell proteins *ccmK1, ccmK2*, and two variants of each of the four genes of the putative high-affinity bicarbonate transporter BCT1, i.e., *cmpA, cmpB, cmpC*, and *cmpD* were extracted from the genomic sequence. All nucleotide sequences were translated into protein sequences and subjected to maximum-likelihood (ML) analysis as previously described (Tang et al., 2022c).

### Gene transcription analysis during CO_2_ shift experiment

A CO_2_ shift experiment was performed to analyze the expression profiles of 10 putative genes responsible for carbon uptake in the A174 strain. As described earlier, the cells of A174 were cultivated in a shaking incubator to induce the formation of biomass pellets of ~1 mm in diameter. The pellets were harvested by filtration, and ~6,000 pellets corresponding to the dry weight of nearly 0.2 g were used for the CO_2_ shift experiment. The strain pellets have been pre-cultured in BG-11 liquid medium under a continuous supply of ambient air for 24 h to adjust to the cultivation mode. All the cell pellets have been harvested by centrifugation at 1,500 × g, washed, and subsequently resuspended in fresh BG-11 medium and grown for 24 h in HC [4% CO_2_ in air (v/v)]. At the end of this cultivation period, ~200 pellets have been collected, flash-frozen in liquid nitrogen, and used for RNA extraction (HC). The remaining cells have been shifted to lower, ambient, carbon environment by replacing the CO_2_-enriched air with ambient air (0.042% CO_2_, defined as low CO_2_, LC). The cultivation in LC lasted for 1 h, and an equal amount of biomass was used for RNA extraction (LC1). The remaining cells were grown for another 23 h, and another batch of cells was used for RNA extraction (LC24). The total RNA from each of the three biological replicate samples was extracted using RNAiso Plus reagent (Takara, Dalian, China) and treated with DNAseI (Thermo Fisher Scientific, Waltham, USA) according to the manufacturer's instructions at the following three time point: HC, LC1, and LC24. RNA concentration and integrity have been assessed with Nanophotometer (Implen, Germany) and agarose gel electrophoresis, respectively.

Real-time quantitative polymerase chain reaction (RT-pPCR) was used to assess 10 genes representing predicted distinct components of the A174 CCM system. The following genes have been selected for the analysis: *ndhF3, ndhF4, cmpA1, cmpA2, bicA, sbtA, rbcL, ccmK1, ccaA1*, and *ccaA2*. The *rpoB* was used as an internal control among other genes based on the expression stability vs. target genes and relatively high Ct value. The oligonucleotides used in the qPCR reaction are summarized in Supplementary Table 3. Reverse transcription was performed with PrimeScript RT reagent Kit (Perfect Real-Time kit, Takara, China). The resultant cDNA was a template for the quantitative estimation of gene expression levels. The quantitative PCR reaction was done with TB Green Premix Ex Taq (Tli RnaseH Plus) (TaKaRa, Dalian, China) in an Applied Biosystems QuantStudio 5 Real-Time PCR System (Thermo Fisher Scientific, Waltham, America) using the following conditions: denaturation at 95°C for 30 s, annealing at 95°C for 5 s, and then 40 cycles of 60°C for 30 s for extension, followed by a melt cycle of 95°C for 15 s, 65°C for 60 s, and 95°C for 15 s. Data were analyzed by the 2^−Δ*ΔCT*^ method, which directly uses the threshold cycle (CT) value to calculate the relative quantification of gene expression.

### Taxonomic evaluation

The taxonomic description followed the recommendations of the Botanical Code, International Code of Nomenclature for Algae, Fungi, and Plants (Shenzhen code) (Turland et al., 2018) and was based on the classification system developed by Komárek et al. (2014).

## Results and discussion

### General genomic characteristics of strain A174

The complete genome of the A174 strain was obtained using a combination of Oxford Nanopore and Illumina sequencing platforms, resulting in a genome coverage of 239 × and 157 × , respectively. No plasmids were identified in the sequencing data by the analysis of closed circular sequences assembled from long-read sequencing. The strain's final genome comprised a single circular chromosome of 5,809,202 bp and exhibited a GC content of 48.6%. The overall analysis of the A174 genome revealed the existence of three identical ribosomal RNA operons, 48 tRNA genes, 1 tmRNA, 4,864 protein-coding sequences, and 4 repeat regions. In total 2,068 protein-coding sequences have been predicted to be hypothetical proteins, consistent with previous findings on thermophilic cyanobacteria (Cheng et al., 2020; Kono et al., 2022).

### Phylogeny reconstruction using 16S rRNA gene

**Figure 1:**
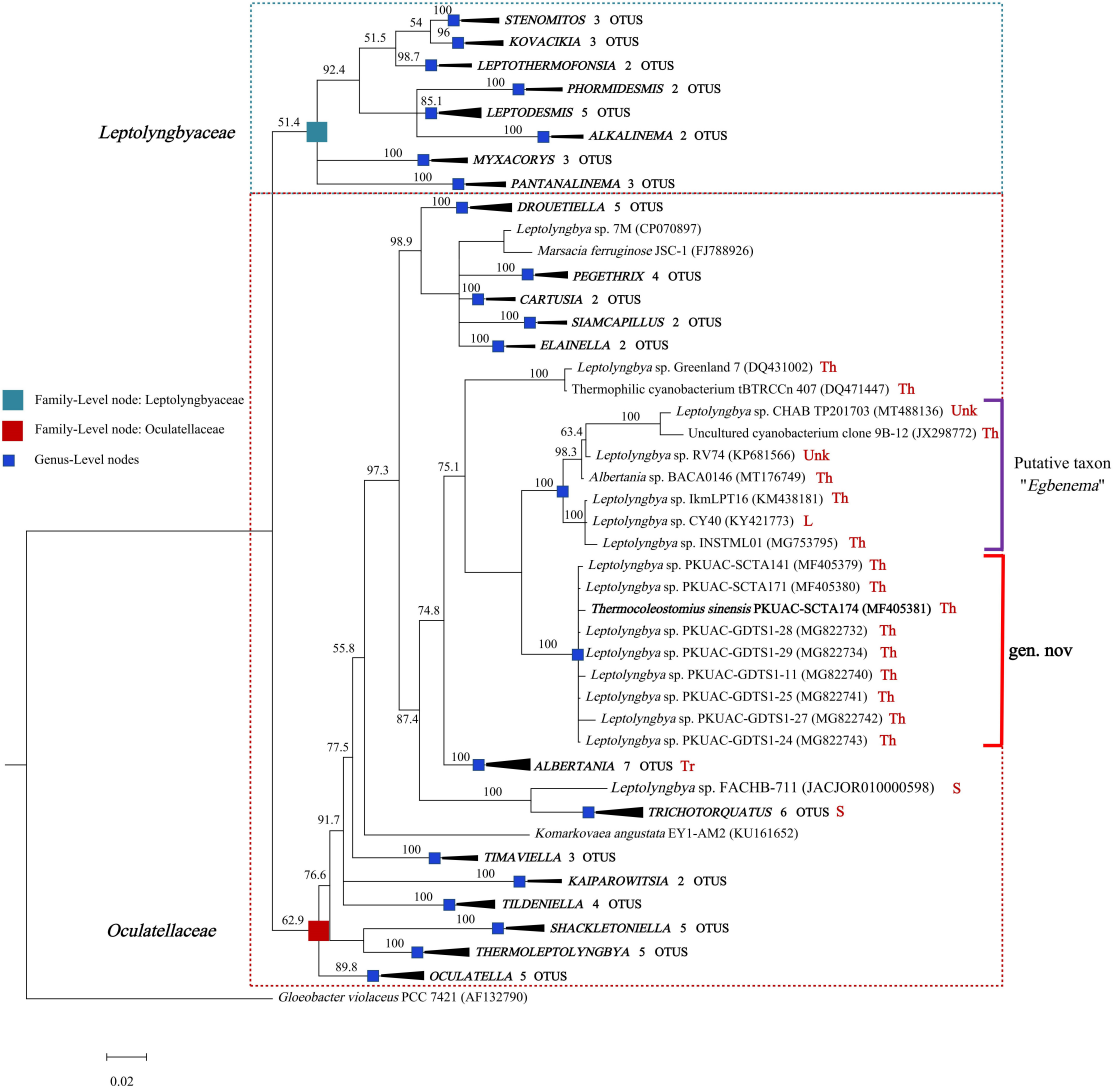
Bayesian inference of 16S rRNA gene sequences representing 91 cyanobacterial strains. Collapsed genera are indicated by black polygons, with a length corresponding to the distance from the most basal sequence to the most diverged sequence of the genus. The complete phylogram refers to Supplementary Figure 1. Posterior probabilities (%) are given above the nodes. The origin (red) of the sequences in the focus clade is provided using the following key: Th, thermal spring; L, lake; S, soil; Tr, terrestrial; Unk, unknown.

The genome of the A174 strain has three identical copies of the ribosomal RNA operon. The sequence of 16S rRNA gene was extracted and aligned with other focus taxa. According to the results of the alignment, 91 cyanobacterial strains' sequences were selected to construct 16S rRNA phylogenetic trees (Figure 1, Supplementary Figures 1, 2). The Bayesian tree inferred from nucleotide sequences in NCBI clearly separated new strains from other well-described genera. The tree was categorized into 22 genera, belonging to the order *Synechococcales* and including families of *Oculatellaceae* and *Leptolyngbyaceae*. The branch containing members of the family *Leptolyngbyaceae* included *Stenomitos* (Shalygin et al., 2020), *Kovacikia* (Miscoe et al., 2016), *Leptothermofonsia* (Tang et al., 2022b), *Phormidesmis* (Komárek et al., 2009), *Leptodesmis* (Raabova et al., 2019), *Alkalinema* (Vaz et al., 2015), *Myxacorys* (Pietrasiak et al., 2019; Soares et al., 2019), and *Pantanalinema* (Vaz et al., 2015). Family *Oculatellaceae* included *Drouetiella* (Mai et al., 2018), *Pegethrix* (Mai et al., 2018), *Cartusia* (Mai et al., 2018), *Siamcapillus* (Tawong et al., 2022), *Elainella* (Jahodárová et al., 2018), *Albertania* (Zammit, 2018), *Trichotorquatus* (Pietrasiak et al., 2021)*, Timaviella* (Sciuto et al., 2017), *Kaiparowitsia* (Mai et al., 2018), *Tildeniella* (Mai et al., 2018; Strunecky et al., 2019), *Shackletoniella* (Strunecky et al., 2019), *Thermoleptolyngbya* (Sciuto and Moro, 2016), and *Oculatella* (Zammit et al., 2012). In addition, *Gloeobacter* genus which has a distinct, independent basal position, was used as an out-group to root the resultant phylogenetic tree accurately.

The position of A174, another two sequences retrieved from the isolates of this spring A141 and A171 and clones isolated from the thermal spring of Huizhou region in China (GDTS1-11, GDTS1-24, GDTS1-25, GDTS1-27, GDTS1-28, GDTS1-29) (Zhang et al., 2019), formed a well-defined clade with high posterior probabilities and 98.96% 16S rRNA sequence identity (Supplementary Table 4) that was clearly separated from other related cyanobacteria. All strains in this clade shared 16S rRNA molecular signature, morphological, and ecological traits. Phylogenetic analysis indicated that the novel clade containing A174-like species is closely related to *Albertania* (93.86%−94.90% 16S rRNA sequence identity) and *Trichotorquatus* (91.59%−92.27% identity) genera already described (Zammit, 2018; Pietrasiak et al., 2021). The closest related clades were occupied by the strains exhibiting *Leptolyngbya*-like (*sensu lato*) morphology such as *Leptolyngbya* sp. IkmLPT16, *Leptolyngbya* sp. CY40, *Leptolyngbya* sp. INSTML01, *Leptolyngbya* sp. RV74, and *Leptolyngbya* sp. CHAB TP201703. The 16S rRNA sequence identity between A174 and the neighboring strains are as follows: *Leptolyngbya* sp. IkmLPT16 (95.36%), *Leptolyngbya* sp. CY40 (95.36%), *Leptolyngbya* sp. INSTML01 (94.9%), *Leptolyngbya* sp. RV74 (95.48%), *Albertania* sp. BACA0146 (95.36%), *Leptolyngbya* sp. CHAB TP201703 (95.36%), and Uncultured cyanobacterium clone 9B-12 (95.24%). All these strains are likely to belong to a recently proposed novel taxon “*Egbenema*” (Akagha, 2022). Meanwhile, there are insufficient data to classify the two more distant strains, a benthic isolate from Artic hot springs of Kap Tobin (Greenland_7) (Roeselers et al., 2007) (94.22% sequence identity) and an unidentified filamentous strain from thermal springs in Jordan (tBTRCCn407) (Bruno et al., 2009) (94.11% sequence identity). This level of sequence identity between the representatives of the two clades is at the border of differentiation of a novel genus (Rodriguez-R et al., 2018) and should be supported with additional analyses. To further classify these strains to their respective taxonomic groups, additional analyses were performed using a polyphasic approach.

### Genome-based analyses

Since 16S rRNA-based taxonomy, based on a single evolutionary marker, has some limitations (Johnson et al., 2019), it is important to supplement it with other methods, especially when borderline values of genus or species delineation are observed. To make the most of the complete genome sequence of the strain and to ascertain the taxonomic position of the clade containing A174, genome-based analyses were employed. In total, three genome-based coefficients were applied to aid the demarcation of the strain: average nucleotide identity (ANI), average amino acid identity (AAI), and digital DNA–DNA hybridization (dDDH). These parameters are presented in Table 1 and Supplementary Table 6. Methods based on ANI and AAI have proven to be useful for identifying and classifying different genera and are widely employed in taxogenomics. The ANI values (< 83%) (Walter et al., 2017) and AAI values (<65%–72%) (Konstantinidis and Tiedje, 2007) are considered accepted values for genus-level discrimination. Meanwhile, two genomes are considered to belong to the same species if both ANI and AAI values between them are equal to or greater than 95% (Jain and Rodriguez, 2018).

**Table 1:** Values of average nucleotide identity (ANI) and average amino acid identity (AAI) between genomes studied.

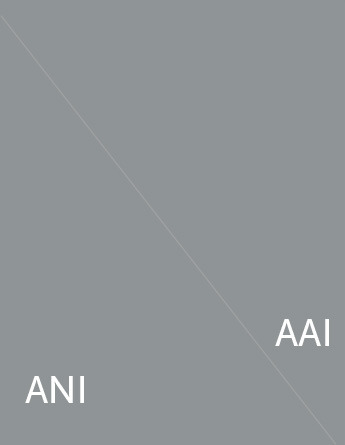	**A174**	**Uher 20002452**	**GSE-PSE-MK54-09C**	**CCNUW1**	**A121**	**7M**	**FACHB-711**	**JSC-1**	**O-77**	**E412**	**FACHB-28**	**LEGE06141**	**CLA17**	**A183**	**UHER199813D**
A174	100.00	65.92	62.58	61.68	63.13	57.71	66.56	67.65	64.94	63.68	65.22	66.22	60.76	65.33	59.13
Uher 20002452	73.64	100.00	60.96	61.41	61.58	56.45	65.32	66.12	63.33	61.65	64.87	64.73	61.94	63.80	58.48
GSE-PSE-MK54-09C	74.13	73.16	100.00	58.41	60.12	52.27	60.93	61.06	64.98	60.21	61.75	62.17	58.82	65.14	58.39
CCNUW1	77.83	75.91	74.45	100.00	66.88	53.22	61.06	61.73	60.62	70.31	62.78	61.91	64.71	61.16	56.92
A121	79.07	73.49	73.74	76.89	100.00	53.89	62.45	62.91	62.77	68.77	62.86	63.07	69.17	63.07	58.03
7M	79.07	75.42	0.00	80.19	80.20	100.00	56.66	83.12	55.22	54.54	55.57	55.59	51.70	55.26	51.11
FACHB-711	75.07	76.09	72.90	76.99	75.35	77.35	100.00	66.49	63.46	62.07	64.99	65.21	61.15	63.69	58.54
JSC-1	75.81	74.34	72.95	76.50	75.01	93.96	75.81	100.00	64.18	63.49	65.14	65.67	60.58	64.54	58.88
O-77	77.57	74.67	74.69	76.14	78.58	78.43	75.70	74.88	100.00	62.74	64.28	64.29	60.41	93.57	58.99
E412	81.81	73.45	75.57	76.48	78.79	78.67	74.86	75.40	79.95	100.00	63.27	63.49	65.06	63.15	58.15
FACHB-28	73.55	76.90	73.24	77.59	74.15	77.17	77.36	75.05	73.99	74.41	100.00	68.09	62.13	64.74	59.26
LEGE06141	75.16	77.69	76.27	78.01	74.73	78.45	77.42	82.74	74.75	76.23	77.68	100.00	61.65	64.57	58.71
CLA17	77.38	79.73	74.46	78.56	75.39	0.00	77.34	73.55	74.83	75.13	77.86	79.73	100.00	60.74	57.24
A183	76.98	74.59	74.95	76.81	77.32	78.64	75.88	75.55	89.97	79.34	74.12	74.44	75.44	100.00	59.23
UHER199813D	72.26	78.06	76.78	73.67	72.76	74.35	75.37	73.54	74.50	72.54	76.51	76.63	80.58	74.53	100.00

The numbers above and below the diagonal indicate the AAI and ANI values (%), respectively.

The two coefficients can be used to verify the taxonomic position of the strains on the phylogenetic trees. The strain A174 has the closest ANI of 81.81%, to the recently sequenced *L. sichuanensis* E412. These parameters are below the level of both species and genus demarcation. The 16S rRNA taxonomy-based analysis revealed that among the strains with whole genomes sequenced, the strain most closely related to A174 was *Leptolyngbya* sp. FACHB-711. The ANI between the two strains was 75.07%, markedly lower than that of E412. Currently, there is a lack of conclusive evidence to determine the exact cause of this phenomenon. There are two non-mutually exclusive hypotheses. One possibility is the convergent evolution of A174 and E412. The two strains could independently evolve similar traits as a result of adapting to similar environments. This hypothesis is supported by the fact that the two strains were found in hot springs of similar temperatures in Kangding County of Ganzi Prefecture, and they both exhibit tolerance to elevated bicarbonate concentrations and display similar nitrogen metabolism. Another possible explanation is horizontal gene transfer. Almost a quarter of the protein-coding genes from the E412 strain was found to have been potentially acquired through this mechanism (Tang et al., 2022b). This finding suggests that the ANI of E412 may have been influenced by the transfer of genetic material from another organism resulting in an aberration of the ANI relationship resulting from the evolution. It should be noted that these explanations are not mutually exclusive, and further research is needed to fully comprehend the complex relationship between the ANIs of these two strains. Meanwhile, the AAI coefficient between the A174 and FACHB-711 was 66.56%. This parameter is at the lower boundary for genus demarcation, indicating slightly closer proximity than that of the *L. sichuanensis* E412 (63.68%).

This combined with the results of 16S rRNA phylogeny suggests that the FACHB-711 strain should be reclassified to the genus *Trichotorquatus*, closely related to the clade containing the A174 strain. ANI and AAI values between A174 and other reference genomes were in the 72–82 and 57–68% range, respectively. Those significant genome-level differences support the delineation of the clade at the genus level. The dDDH parameters further support these findings. The calculated dDDH between the A174 and other focus taxa ranged between 16.80 and 31.70%, significantly below the 70% hybridization threshold for prokaryotic species demarcation.

**Figure 2:**
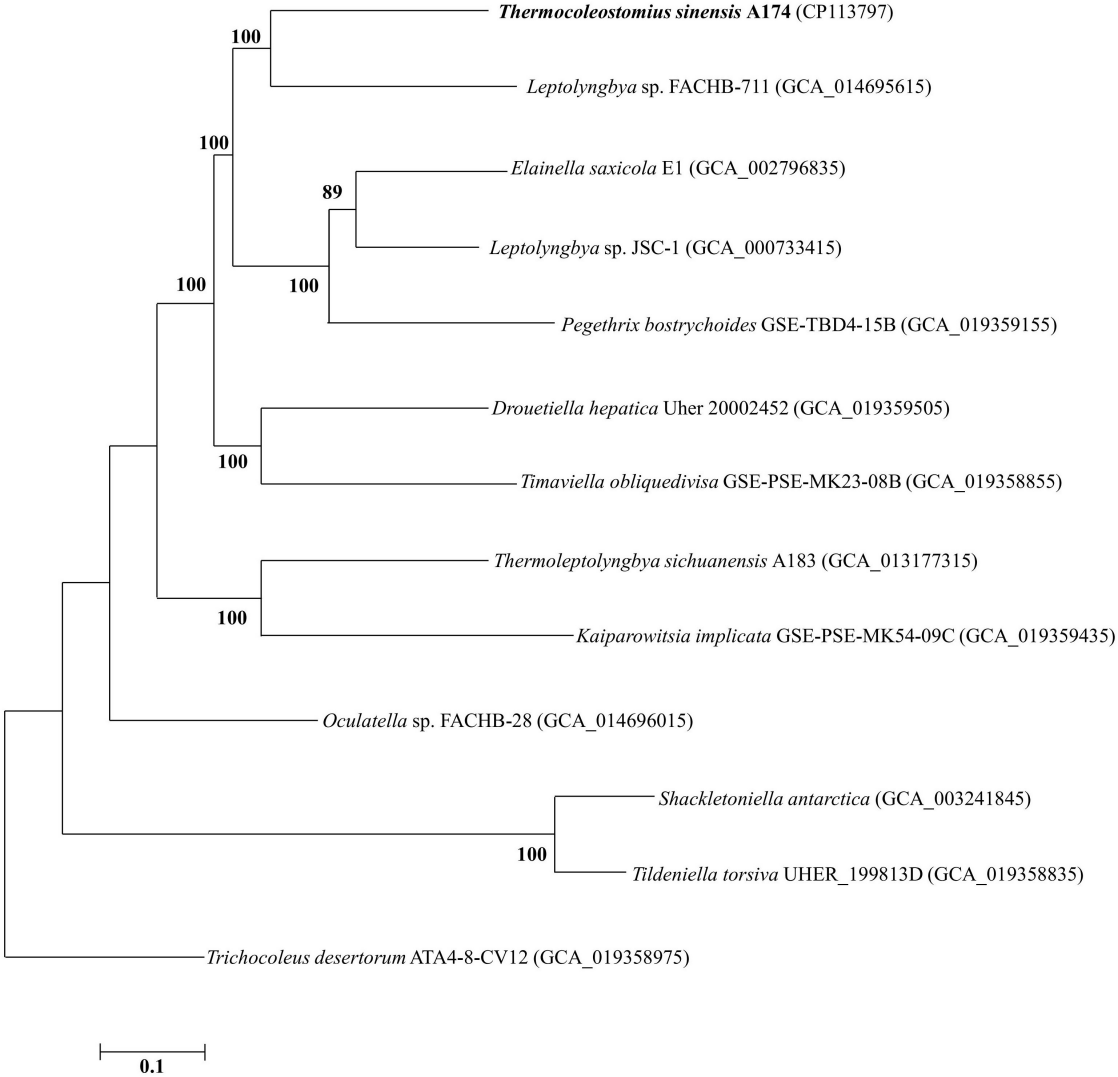
ML phylogenomic tree of a concatenated alignment of 642 single-copy genes shared by all genomes. Strain no. in bold represents the strain identified in this study. Bootstrap values (1,000 replications) are indicated at nodes. Scale bar = 5% substitutions per site.

To further analyze the taxogenomic relationships between the strains, a phylogenomic reconstruction based on single-copy genes shared among the focus taxa with complete genome sequences was performed. A total of 642 single-copy genes were identified to be shared by all the surveyed genomes, generating concatenated alignment with a length of 199,585 aligned amino acid sites. The ML phylogram of the supergene alignment was inferred using the optimal substitution model (LG+F+R5). Each branch of the genomic phylogram is supported by strong bootstrap values (Figure 2), defining tremendous divergence among representative species from each genus. The topology of the resultant tree largely reflects that of the 16S rRNA tree (Figure 1 and Supplementary Figures 1, 2). The unique position of strain A174 confirms its classification to a novel genus. Strain FACHB-711, the closest to the A174 strain with a known genome sequence, is located in a sister clade to strain A174. The genetic distance between those two strains supports their classification to different genera, in line with the 16S rRNA phylogram and the results of genome comparisons, i.e., ANI, AAI, and dDDH. Taken together, *Leptolyngbya* sp. FACHB-711 could be reclassified to the genus *Trichotorquatus*, closely related to the novel genus containing the A174 strain if further genomic studies of well-described *Trichotorquatus* support such findings.

Analyzing all genome-based parameters and phylogenomic reconstruction based on single-copy genes, it can be concluded that the clade containing the A174 strain can be delineated from other taxa as a separate genus. Unfortunately, the scarcity of genomic sequences in the close proximity of the novel clade made its further description using genome-based parameters impossible and more traditional polyphasic methods encompassing detailed analysis of variable regions of ITS, in addition to morphological and habitat characterization need to be employed.

### 16S−23S ITS region and secondary structures

The ITS regions of all three ribosomal operons of strain A174 were extracted from the genome sequence and analyzed along with eight closely related strains regarding sequences and predicted structures of their ITS. Sequence alignment has shown that the three ITS regions of ribosomal operons in A174 were identical. Consequently, only one sequence was analyzed along with the related strains. The highly conserved tRNA regions were removed, and the resulting ITS sequences were processed. When selecting the corresponding operons in related organisms, the specificity of *Trichotorquatus* genus and its operon variability were also considered. Two operons (operons 2 and 3, *sensu* Pietrasiak et al., 2021) of 16S-23S ITS regions were included and compared with the target strains. The length of the resultant sequences varied from 145 to 353 bp (Supplementary Table 5). The variation could be ascribed to the significant difference in variable V2 and V3 helix structure regions. Strains in *Trichotorquatus* genus universally have V3 helixes of ~110 nucleotides. Meanwhile, in other strains, those sequences were either very short (20 nt) or absent.

The focus of the secondary structure analyses was placed on D1–D1′ and boxB regions (Supplementary Figures 3A, B). The evaluation of the predicted structures of the D1–D1′ helix revealed two structural variants that significantly differ in length. The shorter one, encompassing ~56–65 nucleotides sharing a similar overall fold, and a longer one of 80 nucleotides are characteristic for ITS operon 2 of the three main representatives of subclades of *Trichotorquatus*: *Trichotorquatus andrei* CMT-3FSIN-NPC37*, Trichotorquatus ladouxae* WJT66-NPBG9, and *Trichotorquatus maritimus* SMER-A. Additionally, some *Trichotorquatus* species had shorter ITS sequences present in their ITS operon 3. *Trichotorquatus maritimus* had a two nucleotide unilateral bulge when compared to other strains. Meanwhile, *Trichotorquatus* species 5 WJT32-NPBGA lacked bilateral bulge and had three unilateral bulges, which distinguishes it from other structures. It is the most similar in structure to FACHB-711, but the terminal loop of 711 had 4 nucleotides, whereas WJT32-NPBGA had seven nucleotides. One strain, *Albertania* sp. MAR67, had a unique D1–D1′ helix of 111 nucleotides. In addition, except for the similar basal clamp of five base pairs of the D1–D1′ structure, the relatively short LPT16, FACHB-711, and WJT32-NPBGA helices do not have distinct asymmetric loops of multiple nucleotides characteristic for A174 or SA373. The terminal loops of A174 and SA373 were composed of five nucleotides, while that of IkmLPT16 had 12 nucleotides. On the basis of the current, limited description of “*Egbenema*,” “*Egbenema gypsiphila*” species possessed an enlarged terminal loop of 16 nucleotides, which was absent in other strains (Akagha, 2022). The length of the boxB region varied from 35 to 65 bp. Analysis of the boxB region's calculated structures shows similarity of A174 and LPT16 structures. The difference was that the end of the A174 hairpin was a 5-base asymmetric loop, while LPT16 was a 6-base symmetric loop. Meanwhile, *T. maritimus, T. andrei* in operon 2, and *Albertania* sp. MAR67 have shorter helices than helices in other strains, but the first two are more symmetrical. *Albertania* sp. BACA0713 has one more unilateral bulge in helices of similar length. Moreover, comparing the target strain with the putative *Egebenema* taxon, A174 has three asymmetrical additional bilateral bulge structures in BoxB helix, which all putative “*Egbenema*” strains do not have. The BoxB helix in *T. maritimus* in operon 3 has a unique and large unilateral bulge, which is obviously longer than in other strains. In addition, FACHB-711 is identical in structure and sequence to *Trichotorquatus* species 5 WJT32-NPBGA in BoxB helix, reinforcing that FACHB-711 most likely belongs to the *Trichotorquatus* genus.

The combined analysis of the predicted secondary structures of the D1–D1′ (Supplementary Figure 3A) and boxB (Supplementary Figure 3B) regions in the context of 16S rRNA phylogeny (Figure 1, Supplementary Figures 1, 2) and genomic analyses (Figure 2) suggests that the strain FACHB-711 should be reclassified as *Trichotorquatus*. The genus itself is composed of at least five distinctive species including *Trichotorquatus* species 5 (WJT32-NPBGA)*, T. maritimus, Trichotorquatus coquimbo, T. andrei*, and *T. ladouxae*. All these strains were isolated from dryland soils in North and South America indicating that molecular evidence is consistent with their habitat. In addition, there is considerable diversity among the strains containing *Albertania*, and a currently limited amount of molecular data suggests several divergent species within this genus. Finally, the A174 strain belongs to a novel genus different from the putative taxon “*Egbenema*,” *Trichotorquatus*, and *Albertania*.

### Morphological and physiological characteristics of strain A174

**Figure 3:**
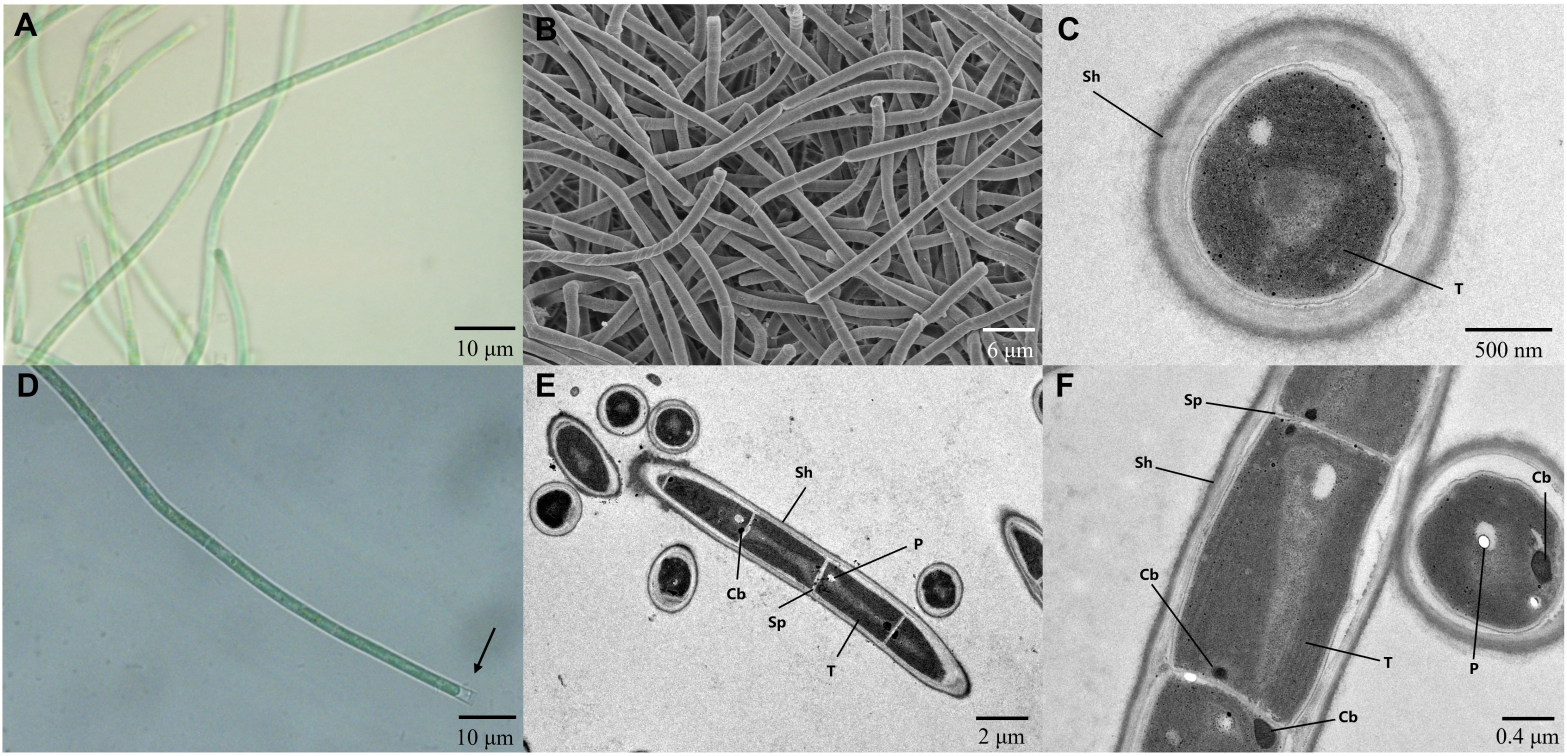
**(A, D)** Light micrographs of *Thermocoleostomius sinensis* sp. A174. Sheath are open at the end (arrow). Scales = 10 μm. **(B)** SEM image of the A174 strain. Scales = 6 μm. **(C, E, F)** TEM micrographs of A174. Short filament showing the round apical cell. A174 cells with carboxysome (Cb), polyphosphate body (P), sheath (Sh), septum (Sp), and thylakoid (T) in the cytoplasm. Magnifications: 400× **(A, D)**, 2,000× **(B)**, 8,000× **(C)**, 1,200× **(E)**, and 5,000× **(F)**.

The strain A174, when grown in the shaking flask cultures, formed very dense, isolated, small trichoid pellets of ~1 mm in diameter containing many entangled filaments. Cells aggregated when reaching a stationary phase in a liquid medium, forming bridges between granules and resulting in a dense mat. Meanwhile, the strain formed dispersed mats or thin laminae on a solid medium. The isolated cyanobacterium was investigated by light microscopy, SEM, and TEM. The image taken by light microscopy (Figures 3A, D) reveals flexuous or bent, sometimes straight trichomes. The strain was phenotypically simple and was composed of elongated cylindrical-shaped cells (Figures 3A–F) surrounded by colorless sheaths that were frequently open at the end, with the terminal cells rounded. False branching was absent. Filaments were solitary with a single trichome per sheath, blue-green in color, accompanied by some minor changes depending on the growth condition or the number of cells. TEM (Figures 3C, F) micrographs indicated that trichomes were cylindrical, composed of isodiametric cells, and the cross-walls of cells had small constrictions. The sheath was thin and colorless, 0.20–0.60 μm thick, usually longer than the trichome, and could be easily observed. Cells are circular in cross section and rectangular in the longitudinal section. Cells of the strain were isodiametric, longer than they were wide (1.5–2.5 μm wide, 2.0–5.8 μm long), with a length-to-width ratio ranging from 1.0 to 2.5 under light microscopy. The number of thylakoids varied from 4 to 6 layers. Thylakoids were arrayed in parallel lines in order at the inner edges of cells (Figures 3C, F). Sheath, septum, phycobilisome, carboxysome, and polyphosphate bodies can also be identified in the cytoplasm (Figures 3C, E, F). The morphological characteristics of other isolates from the same spring, the A141 and A171, were near-identical to the strain A174 (Supplementary Figure 13) and isolates of Huizhou region, i.e., GDTS1–24 and GDTS1–29 (Zhang et al., 2019) confirming the similar morphology of the entire clade.

The overall morphology of the described clade was similar to the previously described cyanobacterium *Albertania skiophila* (Zammit, 2018), with some notable exceptions. Light microscope images revealed that *A. skiophila* frequently exhibited a spiral shape, a feature, which was not observed in A174 and closely related strains. Moreover, *A. skiophila* grown at its physiological conditions exhibited cylindrical trichomes widened at their apices; meanwhile, the A174 was typically thinner than wide. Other strains belonging to *Albertania*, e.g., freshwater *A. alaskaenisis*, were characterized by apparent false branching and sometimes formed characteristic mushroom-like calyptra. These features were not observed in A174. Comparison of strain A174 to *Trichotorquatus* is problematic due to the absence of good-quality electron micrographs, but several distinguishing features can be inferred from light microscopy. *Trichotorquatus* strains described vary in their sheath description, ranging from thick to absent (Table 2). This may be attributed to the different life cycle stages and environmental conditions that these strains exhibited during taking the micrographs. Meanwhile, A174 had a defined colorless sheath under the conditions tested. Cell size, shape, and spacing were also distinguishing factors between these two genera. Moreover, the individual cells of *Trichotorquatus* were easily distinguished on light micrographs, while the cells were not well distinguishable in the A174. The cells of the representatives of a putative taxon “*Egbenema*” were often shorter than the wide based on the available light micrographs (Akagha, 2022). The average length and width were 1.9 μm and 2.2 μm in “*Egbenema aeruginosa*,” which is significantly shorter than the long, cylindrical cells of A174. All “*Egbenema*” strains had visible false branches, and the width and length of each cell were very comparable. Trichomes of “*E. gypsiphila*” were mostly constricted at cross-walls.

**Table 2:** Comparison of morphological features and habitats of *Thermocoleostomius sinensis* and closely related strains.

**Species**	**Morphology**	**Cell width (μm)**	**Cell length (μm)**	**Sheath**	**Thylakoids No**.	**Color**	**Habitat**	**References**
*Thermocoleostomius sinensis* A174	Straight, wavy, unbranched, single trichome per sheath	1.5–2.5	2.0–5.8	Thin, colorless	4–6	Green	Thermal spring	This study
*Leptolyngbya* sp. PKUAC-GDTS1–24/29	Straight, wavy, mostly unbranched, few false branched	ND	ND	Thin, colorless	ND	Blue-green	Thermal spring	Zhang et al., 2019
*Albertania alaskaensis*	Solitary, unbranched in young culture, false branching in old culture	1.8–3.0	1.9–4	Thin, colorless	ND	Bright yellow-green	Willow roots near Meltwater Brook	Strunecky et al., 2019
*Albertania* sp. MAR67	Straight, wavy, unbranched, single trichome per sheath	2.0 ± 0.3/1.5–2.8	2.8 ± 0.5/1.5–4.2	Thin, colorless	ND	Green	Rocks in the Sahara Desert	Mehda et al., 2022
*Albertania skiophila* str. SA373 Zammit 2014	Straight, wavy, coiled, single trichome per sheath	2.0–3.0	2.0–4.0	Thin, colorless, firm, often open at the end	6–10	Green, blue-green	Humid calcareous rock walls, calcareous plasters and mortars in the cave	Zammit, 2018
*Albertania egbensis* N14-MA1	Straight or spiraled, entangled, rare false branching	1.6–2.6	2.0–4.6	Narrow, clear, tightly adherent to the trichomes	ND	Pinkish green	Soils or subaerial surfaces in a tropical climate area	Akagha, 2022
*Albertania latericola* N14-MA3	Straight, entangled, rare false branching	2.0–2.6	2.2–4.6	Narrow, clear, tightly adherent to the trichomes	ND	Pinkish green	Soils or subaerial surfaces in a tropical climate area	Akagha, 2022
*Trichotorquatus maritimus* SMER-A	Solitary, rarely single and double branching, single trichome per sheath	2.1–4.3	0.9–10.0	Thin, sometimes widen or absent	ND	Bright green	Dryland soils	Pietrasiak et al., 2021
*Trichotorquatus coquimbo* ATA2-1-KO25A	Solitary, unbranched, single trichome per sheath	1.8–3.4	1.2–5.6	Sometimes absent, thick when present	ND	Bright green	Dryland soils	Pietrasiak et al., 2021
*Trichotorquatus andrei* WJT9-NPBG15	Solitary, unbranched, single trichome per sheath	1.0–2.8	1.5–5.0	Sometimes absent, thick when present	ND	Bright green	Dryland soils	Pietrasiak et al., 2021
*Trichotorquatus ladouxae* WJT66-NPBG9	Solitary, mostly unbranched, rarely pseudo-branched, single trichome per sheath	2.0–3.6	1.2–4.0	Sometimes absent, thick when present	ND	Bright green	Dryland soils	Pietrasiak et al., 2021
*Trichotorquatus* sp. 5 WJT32-NPBGA	Unbranched, single trichome per sheath	2.0–2.8	1.6–6.0	Sometimes absent, thick when present	ND	Bright green	Dryland soils	Pietrasiak et al., 2021
“*Egbenema aeruginosa* N15-MA6”	Straight, with single trichome per sheath, infrequent false branching	2.0–2.6	1.2–2.8	Narrow, clear, tightly adherent to the trichomes	ND	Bright blue-green	Soils or subaerial surfaces in a tropical climate area	Akagha, 2022
“*Egbenema epilithica* CT225”	Straight and coiled, irregularly entangled or coiled, free or with sheaths	1.6–3.0	1.6–4.0	Narrow, clear, tightly adherent to the trichomes	ND	Blue-green	Soils or subaerial surfaces in a tropical climate area	Akagha, 2022
“*Egbenema aeruginosa”*	Straight and coiled, frequent false branching, free or with sheaths	2.0–2.6	1.8–3.6	Narrow, clear, tightly adherent to the trichomes	ND	Blue-green	Soils or subaerial surfaces in a tropical climate area	Akagha, 2022

The A174 strain was subjected to physiological tests using varying concentrations of bicarbonate and nitrate in different modifications of the BG11 medium (Table 3). It exhibited growth in most nitrogen conditions except for nitrite, and optimal growth was observed at 1.5 g/L nitrate concentration. The strain demonstrated a high tolerance for bicarbonate, with a maximum concentration of 0.7 M. When treated for 21 days with 1 M bicarbonate, the cells turned yellow-green and exhibited poor growth. The strain's tolerance to bicarbonate was attributed to its possession of bicarbonate transporters (BCT1, bicA, and sbtA). Furthermore, the strain displayed phototaxis when grown under directional light (Supplementary Figure 11).

**Table 3:** Physiological characteristic of *Thermocoleostomius sinensis* A174 grown in 45°C 150 μmol m^−2^ s^−1^.

**Dissolved inorganic carbon**		**Nitrogen source**	
**HCO** ${}_{3^{-}}$ **(mol/L)**	**Growth**	**NO** ${}_{3^{-}}$ **(g/L)**	**Growth**
0	+++	0	++
0.1	++	0.075	++
0.3	+++	0.5	++
0.5	+++	1.5	+++
0.7	+	5	++
1.0	–	7.5	+
		1.218 (${\text{NO}}_{2^{-}}$)	–

“–” no growth; “+” poor growth; “++” baseline growth in BG-11; “+++” better growth than baseline.

### Taxonomic position of A174 strain using polyphasic approach

In accordance with contemporary cyanobacterial taxonomy standards, Komárek et al. (2014) have defined the concept of a cyanobacterial genus based on three criteria. These criteria include the following: (i) a distinct taxonomic position with a discernible divergence (of 95% or less 16S rRNA gene sequence similarity) from the nearest sister clade, (ii) unique morphological traits or biological specificity (for example, type of cell division, formation of heterocyte or akinete, etc.) that distinguish it from other genera, and (iii) clear ecological niches that are relevant to the genus. The molecular 16S rRNA gene sequence similarities between the clade of interest and related clades of *Trichotorquatus* and *Albertania* were lower than 95% similarity. Delineation of A174 as a new genus was additionally supported with genomic similarity coefficients, phylogenomics, and secondary structures of D1–D1′ and boxB regions. Analysis of the habitat and morphological features reinforces the molecular evidence. Strains in *Trichotorquatus* genus were isolated from dryland soils in the desert or coastal scrub, which exhibit the sheath with a distinctive collar-like fraying and widening mid-filament. *Albertania skiophila* filaments formed biofilms on the rock surface at the crypt, and MAR67 was collected from gypsum blocks in the hyper-arid Sahara Desert. Meanwhile, Alaskan strains of *Albertania* isolated from willow roots appear unique morphologically with undulating trichomes with perpendicular cells and false branching in old cultures. Both related genera *Trichotorquatus* and *Albertania* have morphological and ecological niche differences that can support the delineation of the thermal clade containing the A174 strain as a new genus.

The exact separation between the clade and adjacent clade, putatively described as “*Egbenema*,” is more problematic due to the lack of sufficient data on the strains that are not a part of this study. Analysis of the three aspects of genus-level delineation: molecular, morphological or physiological characteristics and habitat data, allows for drawing the following conclusions. Based on the 16S rRNA sequence identity between the Chinese strains (A174, A171, GDTS1–24, and GDTS1–29) and sister clade containing “*Egbenema*” (Figure 1), which ranges from 94.9 to 95.48%, indicating that based on the 16S rRNA sequence alone, it is uncertain if strains belong to the same genus. Analysis of the secondary structure of ITS helices D1–D1′ and BoxB reveals that the A174 strain has a more diverse asymmetrical additional bilateral bulge structure. The habitat data (Figure 1 and Table 2) show that strains in the focus clade originate from thermal environments supporting the ecological niche requirement for the genus delineation. Meanwhile, the strains proposed as “*Egbenema”* show diverse habitats. “*Egbenema”* strains were isolated from soil or subaerial samples in a tropical climate area and can survive in perennially wet habitat. There is also a clear morphological difference between A174 clade and putative “*Egbenema*” taxon. The members of A174 clade, contrary to strains described as “*Egbenema*,” had no false branching, and their overall cell shape was different. Cells of the A174 clade are longer than wide, while those of “*Egbenema*” representatives had similar length and width. The terminal cells of the trichomes have also shown different morphologies. The characteristic pellet-forming feature of A174 was not observed in “*Egbenema”* according to the available literature.

To summarize, at least at the level of the strains with morphological data available, the focus clade of this study is consistent in morphology, habitat, and molecular data. A monophyletic cluster, comprising three strains (A171, A174, and A141) isolated from two hot springs in Erdaoqiao (Sichuan Province) and two closely related strains isolated from a single hot spring in Guangdong Province (Zhang et al., 2019), has been included in this clade. These strains exhibit similar morphology and 16S rRNA sequence identity of over 98.9% and have been found to originate from hot springs. This allows us to conclude that they belong to the same species. Consequently, the different morphology combined with very distinct environmental niches and some limited molecular evidence suggests that the clade containing A174 and the putative “*Egbenema”* clade belong to two different genera.

### Genomic outlook on the carbon uptake and concentration in A174 strain

Due to the low solubility of gaseous CO_2_ at elevated temperatures and higher ionic strengths, many thermophilic cyanobacteria deal with higher limitations of inorganic carbon than their mesophilic counterparts (Tang et al., 2022c). In response to this limitation, cyanobacteria frequently form microbial mats in hot springs where the microorganisms are stratified. The top layer is occupied by cyanobacteria that perform photosynthesis and release O_2_, the subsequent layer contains heterotrophs that utilize oxygen for respiration and release CO_2_ that is acquired by the top layer cyanobacteria (Ferris et al., 1997). In the absence of cohabitating organisms, cyanobacteria can also utilize carbon concentration mechanisms (CCM) that employ active transport to increase the amount of carbon dioxide in the carboxysome.

**Figure 4:**
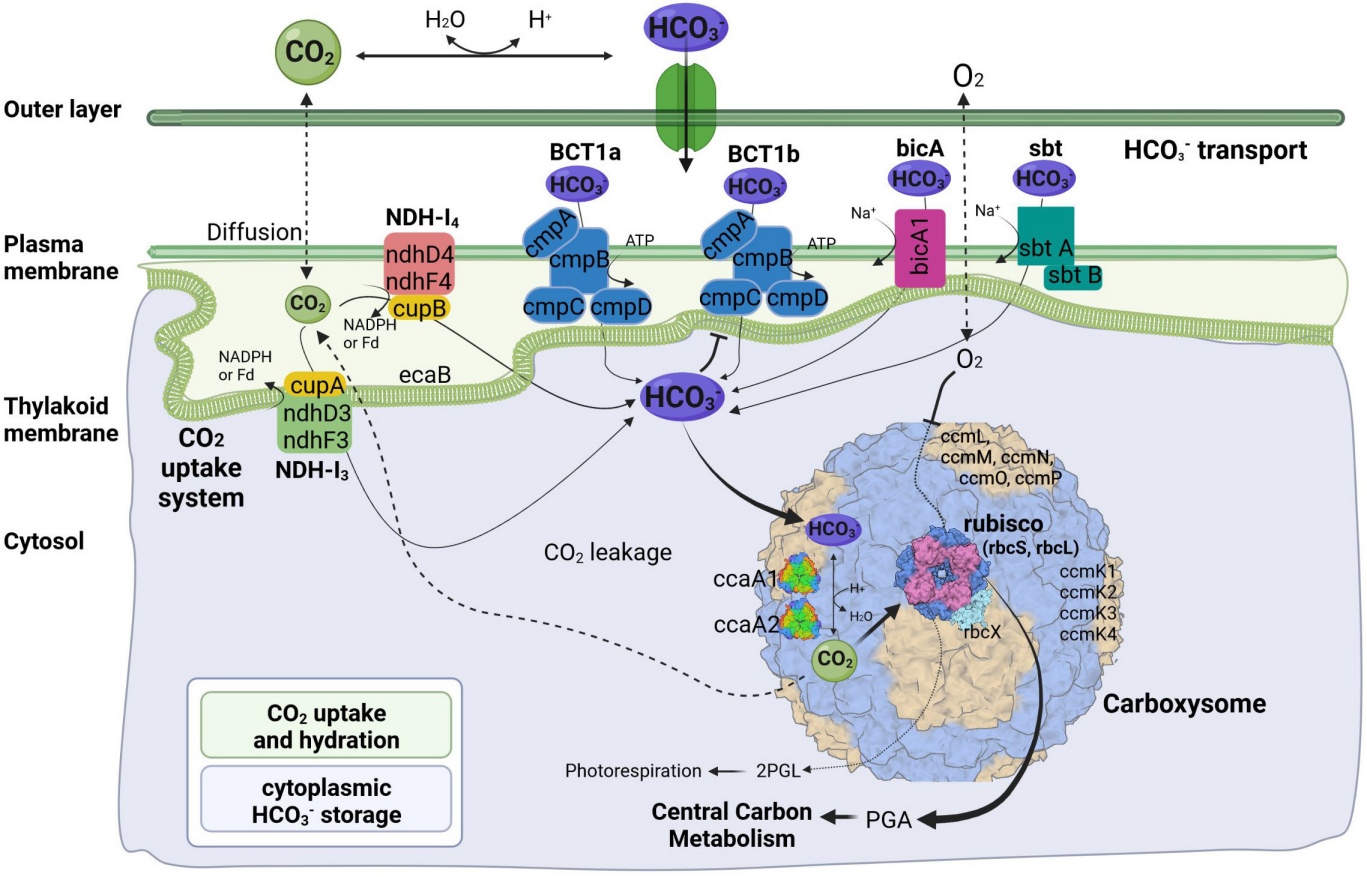
Predicted molecular components of CCM for *Thermocoleostomius sinensis* A174. 2PGL, 2-phosphoglycolate; PGA, phosphoglyceric acid. Three-dimensional structures of proteins visualized using related structures of 2YBV, 6OWF, 5SWC, 5BS1, 6KI1. Figure created with BioRender.com.

**Figure 5:**
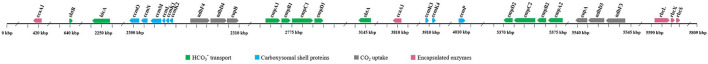
Genomic organization of CCM-related genes for *Thermocoleostomius sinensis* A174. Solid arrow boxes refer to genes and the direction of transcription.

Like their mesophilic counterparts, thermophilic cyanobacteria perform photosynthesis in specialized protein-based structures called carboxysomes, where cytoplasm-stored bicarbonate is transported and converted into gaseous CO_2_ by a carboxysomal carbonic anhydrase to generate high concentration of CO_2_ around the main carboxylating enzyme, Rubisco (Figure 4). The large subunit of this protein, rbcL, has been used to discriminate between α- and β-cyanobacteria (Badger and Price, 2003). The *rbcL* gene of the A174 strain was extracted from the genome sequence and analyzed with other focus taxa using an ML phylogram (Supplementary Figure 4). The results have confirmed that the strain possesses Rubisco 1B form and thus belongs to β-cyanobacteria similarly to terrestrial, freshwater and other thermal strains in accordance with previous studies (Tang et al., 2022c). The gene itself is positioned within an operon containing genes sequentially encoding the Rubisco large subunit (*rbcL*), Rubisco chaperonin (*rbcX*), and Rubisco small subunit (*rbcS*) (Figure 5).

Cyanobacterial CCMs are primarily composed of two main components: inorganic carbon transport system responsible for carbon uptake and carboxysomes responsible for its fixation. Two complexes NDH-I_3_ and NDH-I_4_, responsible for CO_2_ uptake, are ubiquitous in β-cyanobacteria, but there exists a significant diversity in the bicarbonate uptake systems (Price et al., 2008). Analysis of genomic components of the A174 strain reveals a similar pattern. The strain possesses a full complement of genes encoding NDH-I_3_ (low-CO_2_ inducible, high-affinity CO_2_ uptake system) and NDH-I_4_ (constitutive, low-affinity CO_2_ uptake system). The amino acid sequence identity to the orthologous proteins from a mesophilic reference strain *Synechocystis* PCC 6803 of the constitutive ndhD4, ndhF4, cupB, and inducible ndhD3, and ndhF3 components varies from 55.8% (ndhF4) to 62.1% (cupB). Meanwhile, the carbonic anhydrase cupA associated with low-CO_2_ inducible ndhD3 and ndhF3 is more conserved and exhibits an identity of 83.4%. These findings are in accordance with our previous findings on thermophilic strains (Tang et al., 2022c).

The genomic makeup of A174 bicarbonate transport system is diverse compared to other thermophilic strains and most similar to *Leptolyngbya* JSC-1 and *Thermoleptolyngbya* O-77 (Tang et al., 2022c). The strain shows a repertoire of known bicarbonate transporters: BCT1, bicA, and sbtA. Based on the genomic information alone, there is uncertainty about the number of ATP-dependent inducible high-affinity ${\text{HCO}}_{3}^{-}$ transporters, BCT1. There are two regions of high homology to the BCT1 transporters of model strains *Synechocystis* PCC6803 and *Synechococcus* PCC7942 positioned in forms discrete operons at 2.77 and 5.37 Mbp (Figure 5). Both comprise four polypeptides cmpA-D, consistent with other thermophilic cyanobacteria (Tang et al., 2022c). The two putative bicarbonate transporters are the members of two distinct clades (Supplementary Figures 6–9), and their transcription profiles are further analyzed in the CO_2_ shift experiment. In addition to BCT1, the strain possesses two genes of the sbt, the least abundant bicarbonate transporter in thermophilic cyanobacteria, *sbtA* and *sbtB*. The two copies are dispersed in distant loci of the genome. Finally, the A174 strain possesses a single bicA transporter (bicA1), similar to most thermophilic cyanobacteria except for *Thermoleptolyngbya* A183 and *Leptothermofonsia* E412, which have two gene copies of such transporter, *bicA1* and *bicA2*.

Carboxysomes are evolutionarily conserved protein structures that facilitate efficient carbon fixation in cyanobacteria. Carboxysome structures comprise a proteinaceous shell and two encapsulated enzymes, the main carboxylating protein Rubisco and carboxysomal carbonic anhydrase that transforms the soluble bicarbonate into gaseous CO_2_ (Figure 4). Among β-cyanobacteria, the typical structure of shell proteins comprises ccmK-P proteins. Typically, *ccmKLMNO* genes form a discrete operon, and *ccmP* is found in a separate genomic locus. This arrangement is conserved in A174. The strain contains four *ccmK* genes, namely *ccmK1, K2, K3*, and *K4*. The genes coding for two main structural proteins *ccmK1, K2* (Cai et al., 2016), are conserved with other cyanobacteria but do not always cluster with their other thermophilic orthologs on the phylogenetic tree (Supplementary Figure 5). Meanwhile, the sequence of the remaining auxiliary shell proteins ccmK3 and K4 shows the highest similarity to the orthologous thermophilic proteins of *Leptolyngbya* JSC-1. When it comes to encapsulated enzymes, *rbcL* is clearly positioned within the thermophilic cluster of Rubisco but exhibits a relatively diverse sequence compared to other thermophilic cyanobacteria (Supplementary Figure 4) and 96.8% sequence identity to the enzyme of *Leptothermofonsia* E412. Interestingly, there appear to be two copies of carboxysomal carbonic anhydrase *ccaA* dispersed in two loci of the A174 genome, again highlighting higher diversity of strain's CCM than most other thermophilic cyanobacteria. Alignment of the two sequences reveals that the second copy of the gene is ~30% longer than the first (Supplementary Figure 10). More detailed studies will be required to validate the biological function of both carbonic anhydrases *in vivo*.

### Gene expression profiles of the A174 strain during CO_2_ shift experiment

**Figure 6:**
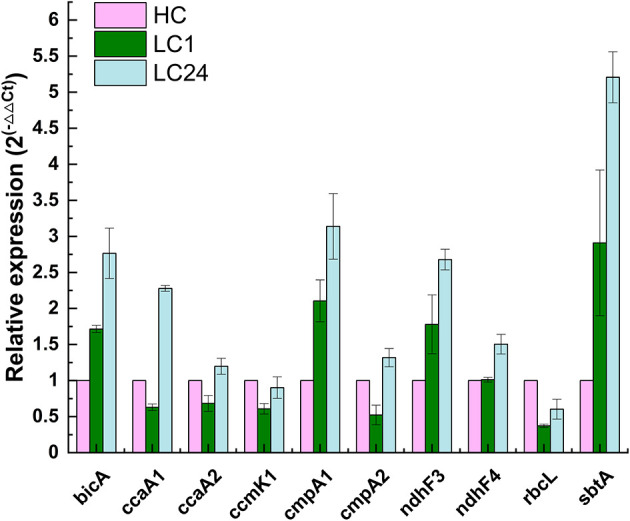
Relative changes in transcript abundance of *ndh*F3, *ndh*F4, *cmp*A1, *cmp*A2, *bic*A, *sbt*A, *rbc*L, *ccm*K1, *cca*A1, and *cca*A2 genes of *Thermocoleostomius sinensis* A174 cells during CO_2_ shift experiment assessed with RT-qPCR. HC indicates high carbon for 24 h (4% CO_2_), LC1 (1 h of carbon limitation), LC24 (24 h of carbon limitation). The data have been normalized to the reference gene *rpo*B and HC conditions.

The CO_2_ shift experiment was performed to check the relative transcription levels of the A174 strain in response to carbon limitation. The cells were initially cultured in a high-carbon environment for 24 h (4% CO_2_ in air) and were subsequently shifted to ambient air. The gene expressions of various bioinformatically identified genes involved in carbon uptake and conversion, including *bicA, ccaA1, ccaA2, cmpA1, cmpA2, ndhF3, ndhF4, rbcL*, and *sbtA*, were subsequently analyzed at two different time point: 1 h and 24 h after the shift. The data were normalized first to the relative abundance of the *rpoB* and subsequently to the transcript abundance under a carbon-replete environment (HC) and are presented in Figure 6.

The results showed that genes related to both low-affinity and high-affinity uptake systems responded positively to carbon limitation but in different magnitudes. NDH-I_3_, the high-affinity system for gaseous CO_2_ uptake, was found to be the dominant system in A174, with 2- to 3-fold increases in expression levels after 1 and 24 h of carbon limitation, respectively. These findings are in line with those for *Synechocystis* PCC6803 (McGinn et al., 2003), where the *ndhF3* gene is undergoing significant upregulation, and *ndhF4* is slightly downregulated during the CO_2_ shift experiment. Similarly, among bicarbonate uptake systems, the transcript levels of high-affinity systems were higher than those of low-affinity ones. The gene expression level of *sbtA* was found to have increased the most compared to other transcripts assessed. Interestingly, the two putative *cmpA* genes exhibited different behaviors in response to carbon limitation, highlighting the need for further function loss studies when appropriate molecular tools become available.

Carboxysome proteins such as shell protein *ccmK1* and two encapsulated enzymes Rubisco (*rbcL*) and carbonic anhydrase *ccaA2* all show a similar pattern of transcription, with a relative drop in gene expression levels after 1 h of carbon limitation, followed by a recovery to baseline levels after 24 h, likely due to the sufficient supply of carbon by the transport components of the CCM system. Interestingly, the second of the carbonic anhydrases, *ccaA1*, exhibited markedly higher expression levels, suggesting a possible different cellular localization than suggested by bioinformatic analyses. Further functional studies are required to validate the function of both putative carbonic anhydrases on the carbon fixation of the A174 strain.

## Conclusion

In this manuscript, we have characterized, using a polyphasic approach, a novel hot-spring cyanobacterium strain A174 as a representative of a novel taxon of thermophilic cyanobacteria. The subject of this study was capable of growth at 50°C and 0.7M bicarbonate. The combination of phylogenetic, taxogenomic, morphological, habitat, and physiological data suggests that the strain could belong to a novel genus of thermophilic cyanobacteria. Based on the results of 16S rRNA phylogeny, comparative genomics, secondary structures of 16S-23S ITS, and strain's distinct morphology to *Albertania, Trichotorquatus*, and recently proposed “*Egbenema”* support the delineation of the clade. Based on those data, we proposed a new genus *T. sinensis* as a best-described representative of this taxon and proposed its delineation within the family *Oculatellaceae*. Additionally, the inorganic carbon uptake and concentration genes have been extracted from the assembled high-quality genome sequence and analyzed. The results have shown that the strain firmly belongs to the β-cyanobacteria and has a diverse array of bicarbonate uptake proteins encompassing bicA, sbtA, and at least one variant of BCT1, and two carbonic anhydrases ccaA. The results of gene expression studies under CO_2_ limitation revealed that all the bicarbonate transporters, bicA, sbtA, and BCT1 are likely to respond to carbon limitation but at different degrees, and the increase of transcript abundance in NDH-I_3_ is higher than that of NDH-I_4_, consistently with mesophilic counterparts.

## Taxonomic treatment and description of *Thermocoleostomius sinensis* Daroch, Jiang, Tang et al. gen. et sp. nov.

The taxonomic classification methodology used for the strain proposed in this study follows the polyphasic approach as described by Komárek et al. (2014). The taxon description was consistent with the prescriptions of the International Code of Nomenclature for Algae, Fungi, and Plants (Shenzhen code) (Turland et al., 2018).

**Phylum**: Cyanobacteria

**Order**: Synechococcales

**Family**: *Oculatellaceae*

**Genus**: *Thermocoleostomius*, gen. nov.

***Description**:* Filamentous cyanobacteria, forming small pellets and radiating outward, colonies intertwined, forming green and compact biofilms and new pellets. Filaments flexuous, wavy or bent, occasionally straight trichomes. Filaments with a single trichome per sheath, no false branching. Sheath frequently open at the trichome termini. Cylindrical trichomes, cross-walls of cells with minor constrictions, cells constricted toward the end. Cells variable in shape, mostly isodiametric to longer than wide, with parietal thylakoids. Apical cell rounded, shorter on average than regular vegetative cells, morphologically similar to regular cells.

***Etymology**:* “Thermo” similar to thermophilic (high temperature tolerant), “coleo” genus epithet derived from the Greek word koleos meaning sheath or scabbard, “stomius” genus epithet derived from Greek word meaning mouth, outlet, indicating that the sheath of the strain is frequently open.

***Type species:*** *Thermocoleostomius sinensis*.

### *Thermocoleostomius sinensis* Daroch, Jiang, Tang et al. gen. et. sp. nov.

***Diagnosis***: Differing from other species of the genus based on the 16S rRNA sequence identity.

***Description**:* Filamentous cyanobacteria (Figures 3A–F), in laboratory cultures, originating from small pellets and radiating outward in diverse directions (Supplementary Figure 14), tiny colonies intertwined and connected, forming green and compact biofilms, appearance of new pellets on solid agar plates (Supplementary Figure 11). Filaments flexuous, wavy or bent, occasionally straight trichomes (Figures 3A, D). Filaments with a single trichome per sheath, no false branching (Figures 3A–F). Sheath frequently open at the trichome termini (Figure 3D). Cylindrical trichomes, cross-walls of cells with minor constrictions, cells slightly constricted toward the end, bright green in healthy cultures, yellow in senescent cultures or in a high light environment. Cells variable in shape, mostly isodiametric to longer than wide (Figures 3A–F), length-to-width ratio 0.8:2.3. Cells size 1.5–2.5 μm long, 2.0–5.8 μm wide, parietal thylakoids arrayed in parallel lines (Figures 3C, E, F). Apical cell rounded, shorter on average than regular vegetative cells, morphologically similar to regular cells (Figure 3E). Strain capable of growth in standard BG11 medium to a maximum temperature of 50°C and medium with 0.7 M NaHCO_3_. Successful cryopreservation of the strain in 10% DMSO for over 2 years. Phototaxis observed (Supplementary Figure 11).

***Etymology:*** “*sinensis*” species epithet derives from the fact that all representatives of the genus have been isolated from various provinces of China.

***Type locality*****:** Thermal spring. Erdaoqiao village in Ganzi Prefecture of Sichuan Province, China.

***Ecology of type locality:*** The sample occurred as a light green biofilm deposited on the surface of calcareous sinter (Supplementary Figure 12).

***Habitat*****:** Thermal spring in Ganzi Prefecture of Sichuan Province, China (30°05′14″N, 101°56′55″E).

***Holotype here designated:*** The dried inactive holotype was deposited in the Herbarium of North Minzu University with the voucher number: NMU00174 (contact: Lei Zhang, zhangsanshi-0319@163.com).

***Reference strain***: The culture *of Thermocoleostomius sinensis* Daroch, Jiang, Tang et al. gen. et sp. nov. was initially denoted and deposited in Peking University Algae Collection as PKUAC-SCTA174, and it also has been deposited in the Freshwater Algae Culture Collection at the Institute of Hydrobiology (FACHB) as *Oculatellaceae* sp. FACHB-3572 after identification and authentication based on the full-length sequencing of the 16S rRNA gene along with folding of the secondary structures of the 16S−23S ITS region.

## Data availability statement

The datasets presented in this study can be found in online repositories. The names of the repository/repositories and accession number(s) can be found in the article/Supplementary material.

## Author contributions

YJ: formal analysis, software, investigation, data curation, visualization, writing—original draft, and writing—reviewing and editing. JT: conceptualization, methodology, validation, formal analysis, investigation, data curation, writing—original draft, writing—reviewing and editing, visualization, supervision, project administration, and funding acquisition. MD: conceptualization, methodology, resources, data curation, writing—original draft, writing—reviewing and editing, supervision, project administration, and funding acquisition.
