# Supplementary material for: Polyphasic characterization of a novel hot-spring cyanobacterium Thermocoleostomius sinensis gen et sp. nov. and genomic insights into its carbon concentration mechanism
Source: Front Microbiol. 2023 Jul 26;14:1176500. doi: 10.3389/fmicb.2023.1176500 (PMC10410155; doi:10.3389/fmicb.2023.1176500)
Supplement: Supplementary file 1 [file Data_Sheet_1.PDF]

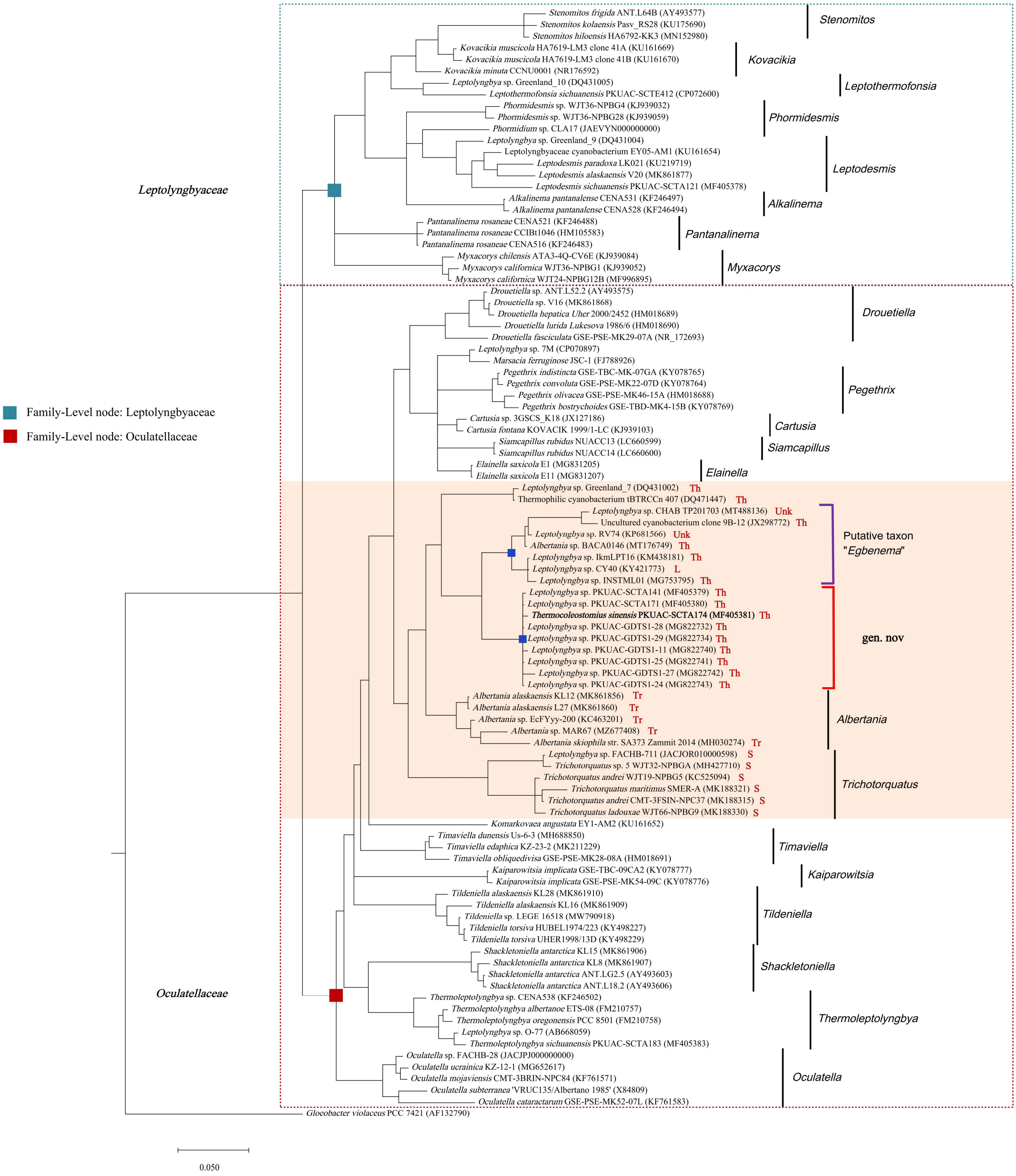

Supplementary Figure 1 Bayesian inference of 16S rRNA gene sequences representing 91 cyanobacterial strains. Posterior probabilities (%) are given above the nodes. The origin (red) of the sequences in the focus clade is provided using the following key: Th, thermal spring; L, lake; S, soil; Tr, terrestrial; Unk, Unknown.

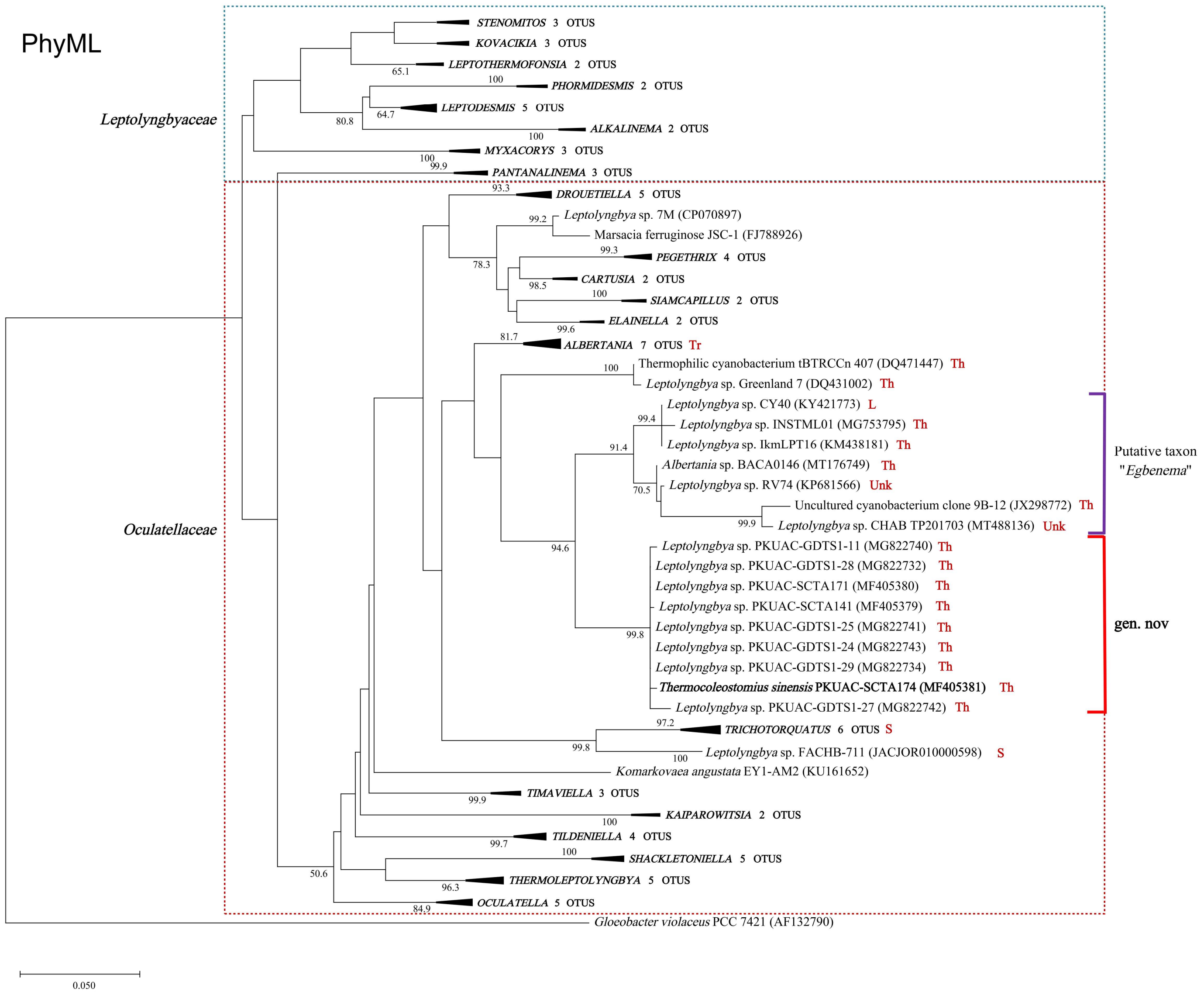

Supplementary Figure 2 PhyML inference of 16S rRNA gene sequences representing 91 cyanobacterial strains. Collapsed genera are indicated by black polygons, with a length corresponding to the distance from the most basal sequence to the most diverged sequence of the genus. Posterior probabilities (%) are given above the nodes. The origin (red) of the sequences in the focus clade is provided using the following key: Th, thermal spring; L, lake; S, soil; Tr, terrestrial; Unk, Unknown.

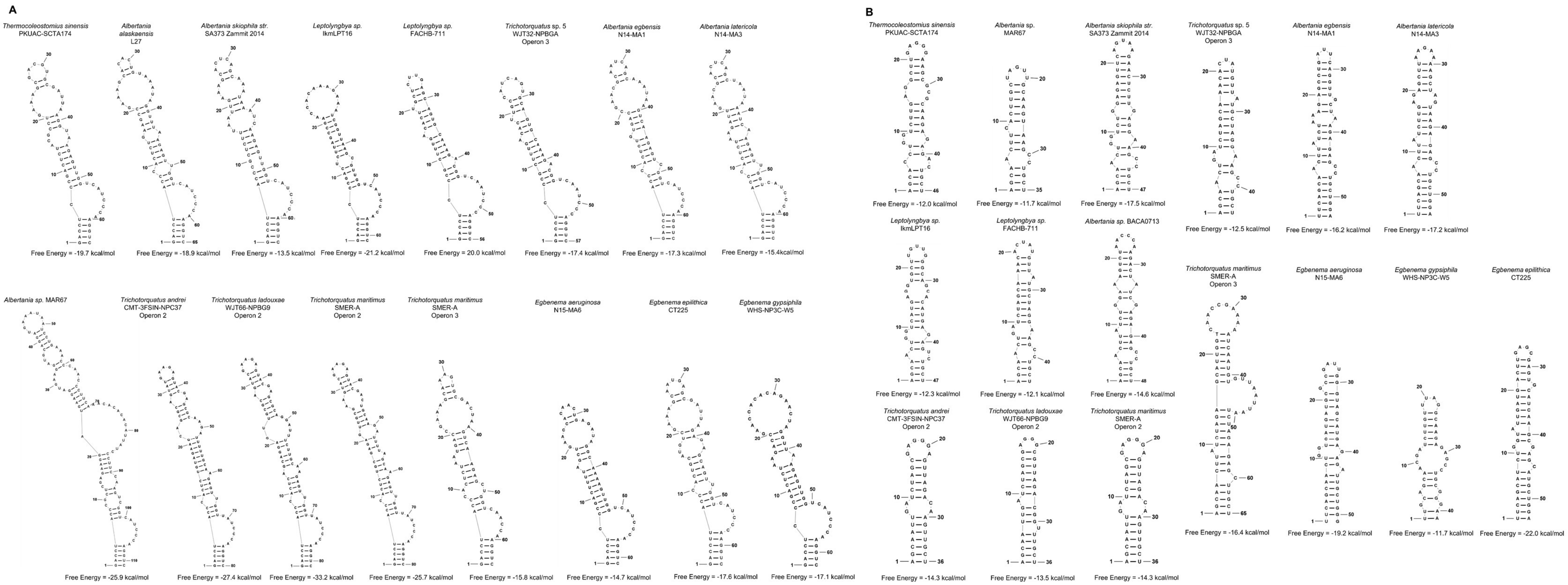

Supplementary Figure 3 Hypothetical secondary structures of D1-D1' helix (**A**), boxB (**B**) of 16S-23S ITS of representative species from family Oculatellaceae, including *Thermocoleostomius sinensis* A174.

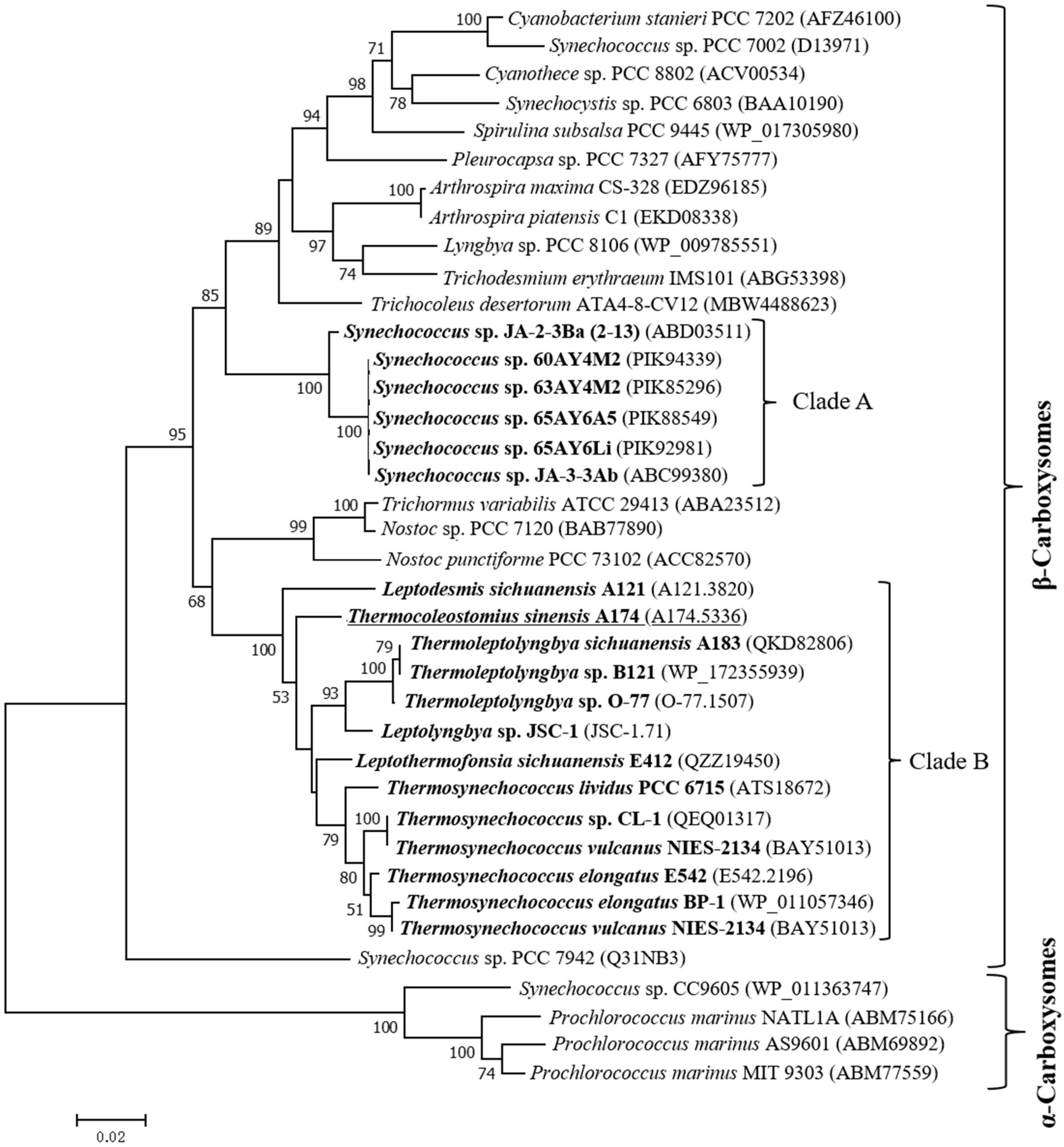

Supplementary Figure 4 Phylogenetic inference of Rubisco large subunit protein sequences. The thermophilic cyanobacteria are indicated in bold. Main subject of the study, the strain A174, is additionally underlined. The accession numbers underscored refers to the gene IDs in Supplementary Table 1 of this study and Supplementary Table 1 of the reference Tang et al. 2022 doi: 10.3389/fmicb.2022.876272.
Only bootstrap values > 50% are indicated at nodes. Scale bar = 1% substitutions per site.

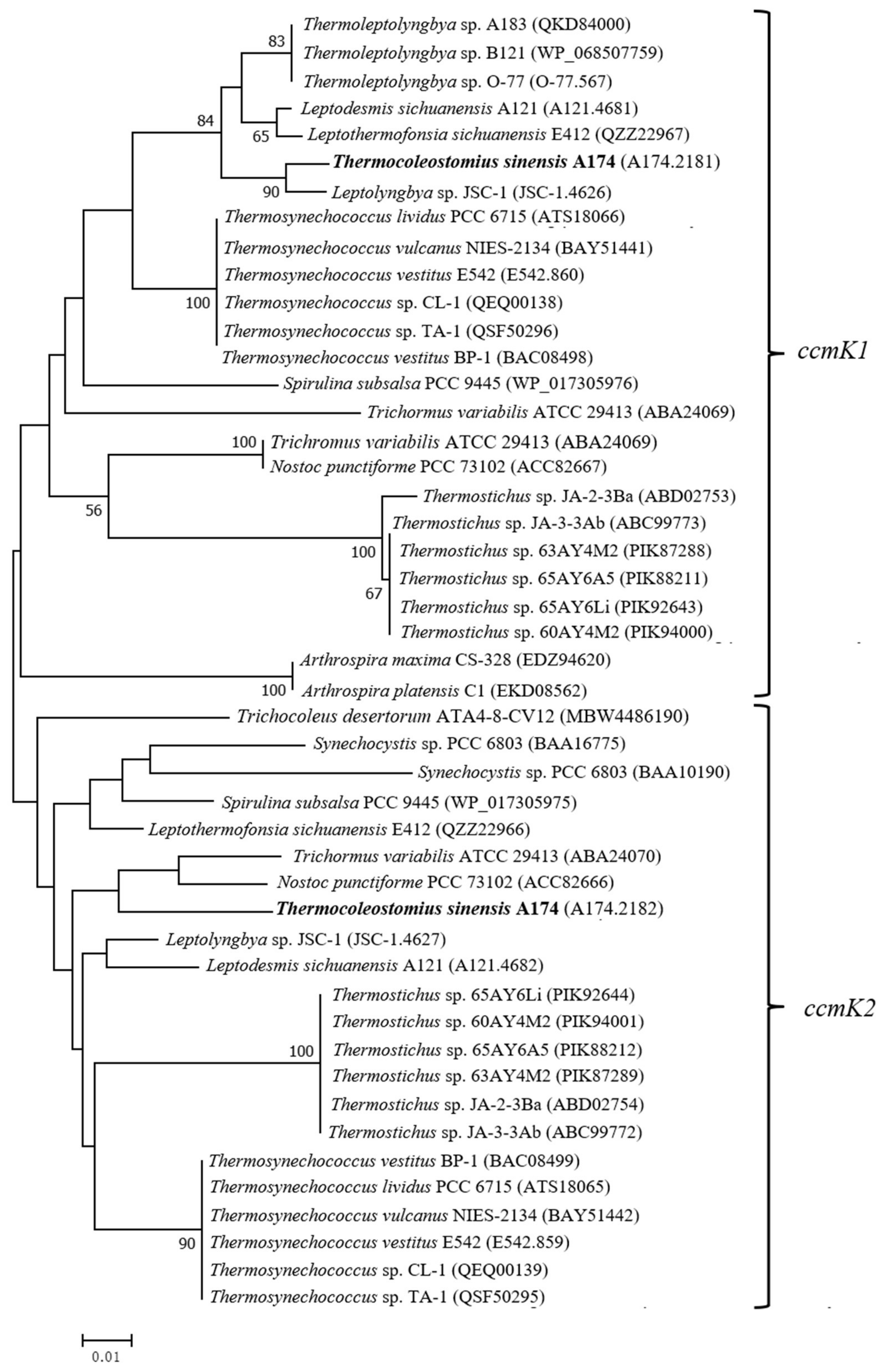

Supplementary Figure 5 Phylogenetic inference of ccmK1 and ccmK2 carboxysome shell protein sequences. A174 strain is marked in bold. The accession numbers underscored refers to the gene IDs in Supplementary Table 1 of this study and Supplementary Table 1 of the reference Tang et al. 2022 doi: 10.3389/fmicb.2022.876272.

Only bootstrap values > 50% are indicated at nodes. Scale bar = 1% substitutions per site.

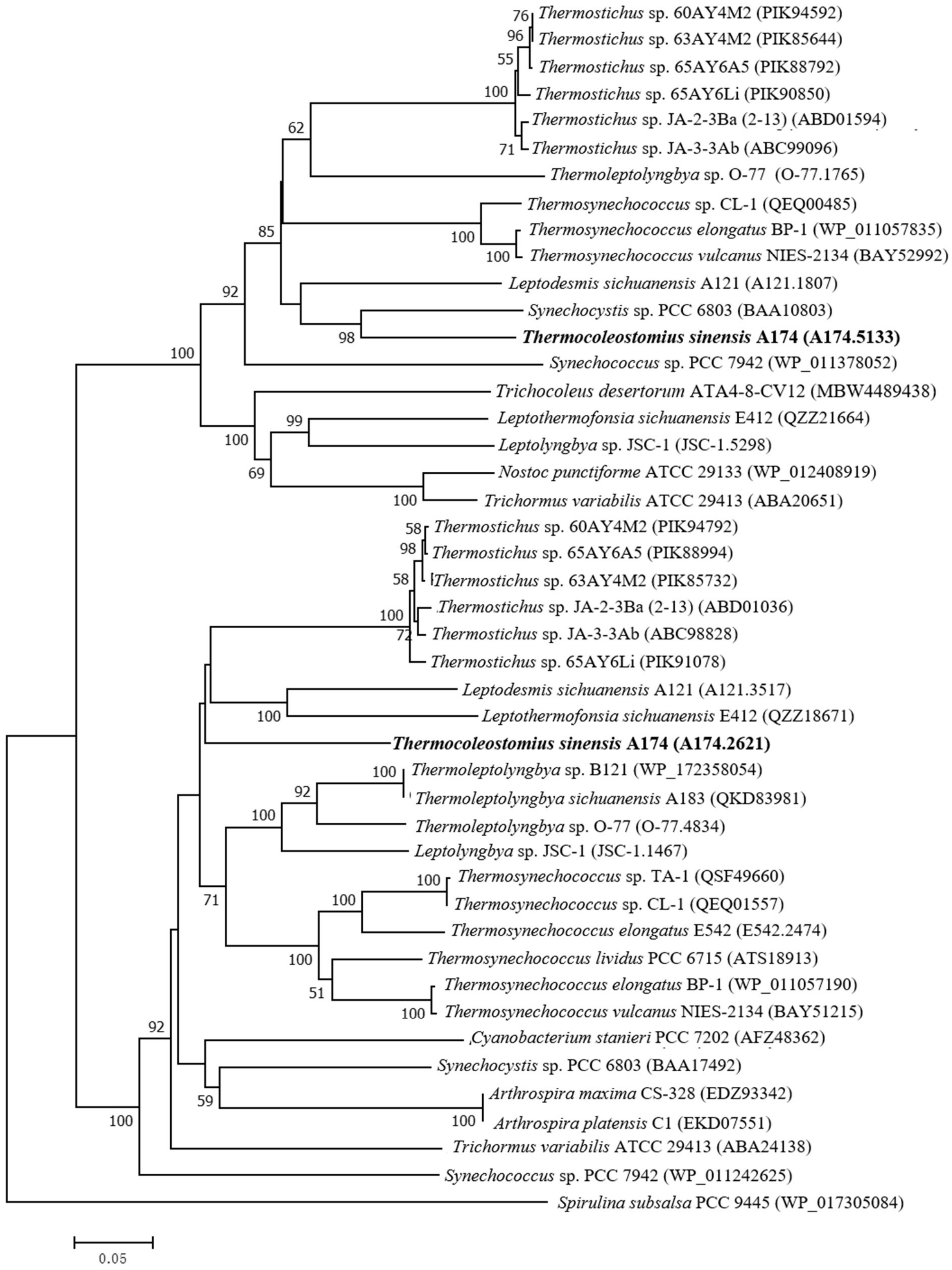

Supplementary Figure 6 Phylogenetic inference of variants of putative *cmpA* gene protein sequences. The accession numbers underscored refers to the gene IDs in Supplementary Table 1 of this study and Supplementary Table 1 of the reference Tang et al. 2022 doi: 10.3389/fmicb.2022.876272.
Only bootstrap values > 50% are indicated at nodes. Scale bar = 1% substitutions per site.

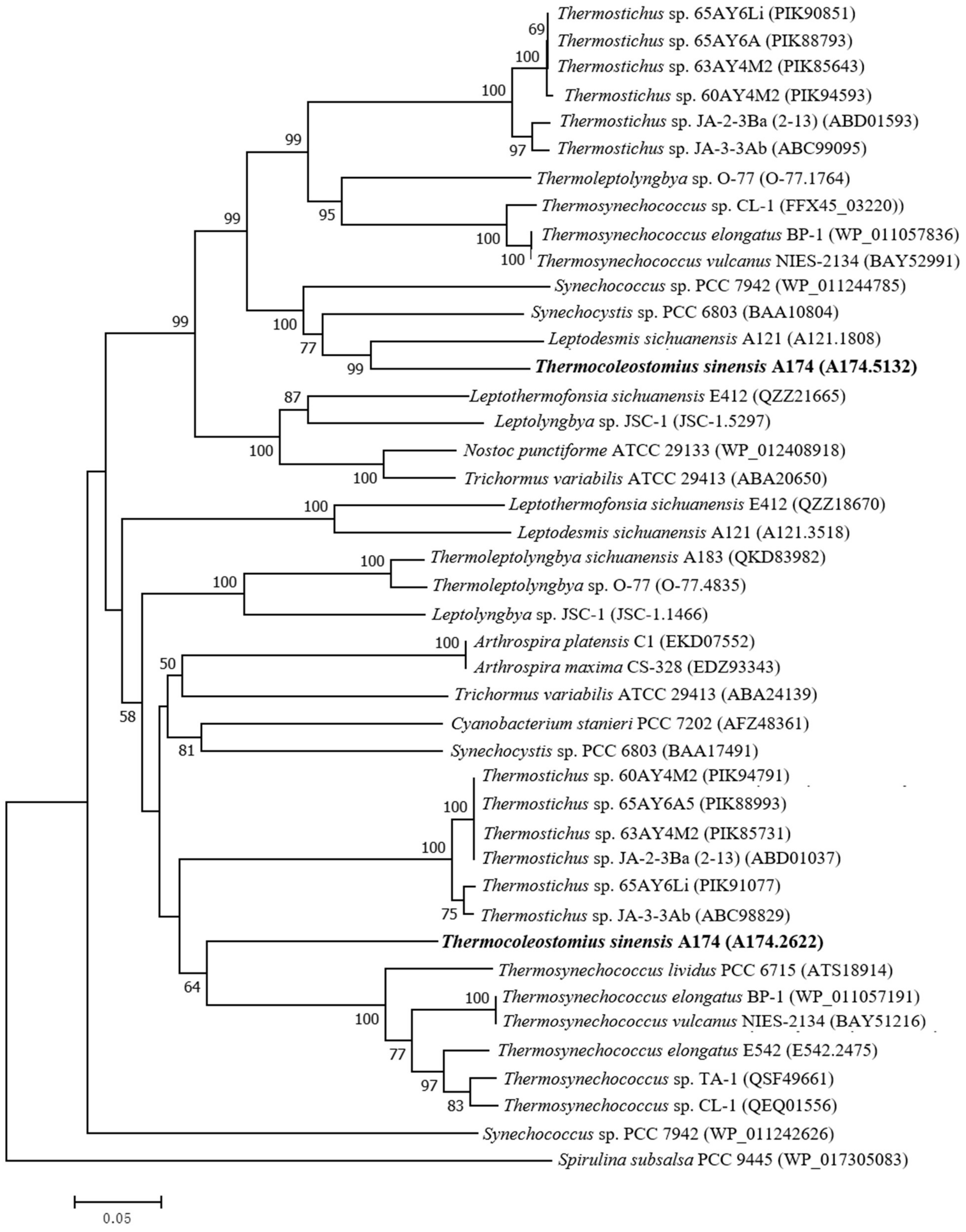

Supplementary Figure 7 Phylogenetic inference of variants of putative *cmpB* gene protein sequences. The accession numbers underscored refers to the gene IDs in Supplementary Table 1 of this study and Supplementary Table 1 of the reference Tang et al. 2022 doi: 10.3389/fmicb.2022.876272.
Only bootstrap values > 50% are indicated at nodes. Scale bar = 1% substitutions per site.

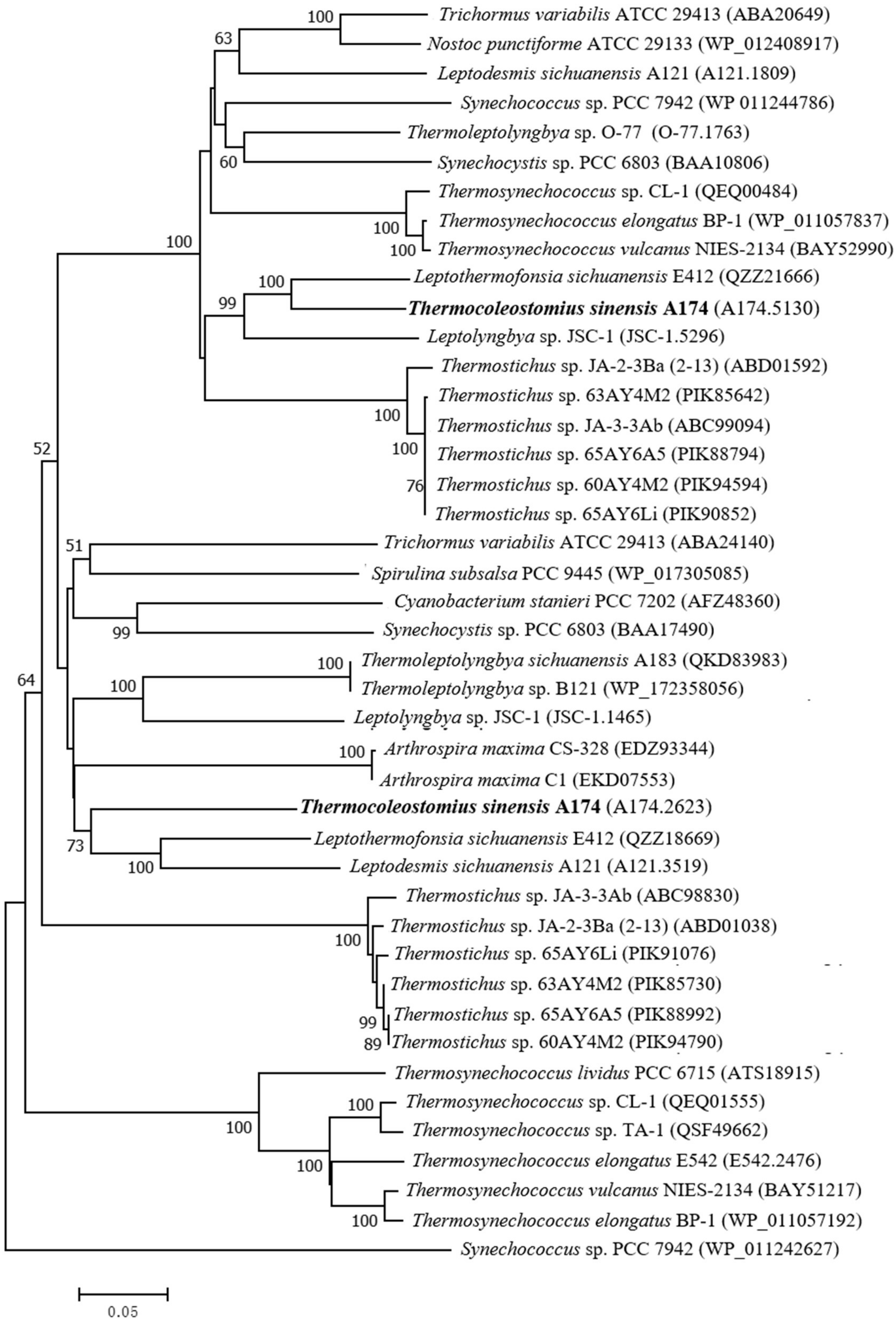

Supplementary Figure 8 Phylogenetic inference of variants of putative *cmpC* gene protein sequences. The accession numbers underscored refers to the gene IDs in Supplementary Table 1 of this study and Supplementary Table 1 of the reference Tang et al. 2022 doi: 10.3389/fmicb.2022.876272.
Only bootstrap values > 50% are indicated at nodes. Scale bar = 1% substitutions per site.

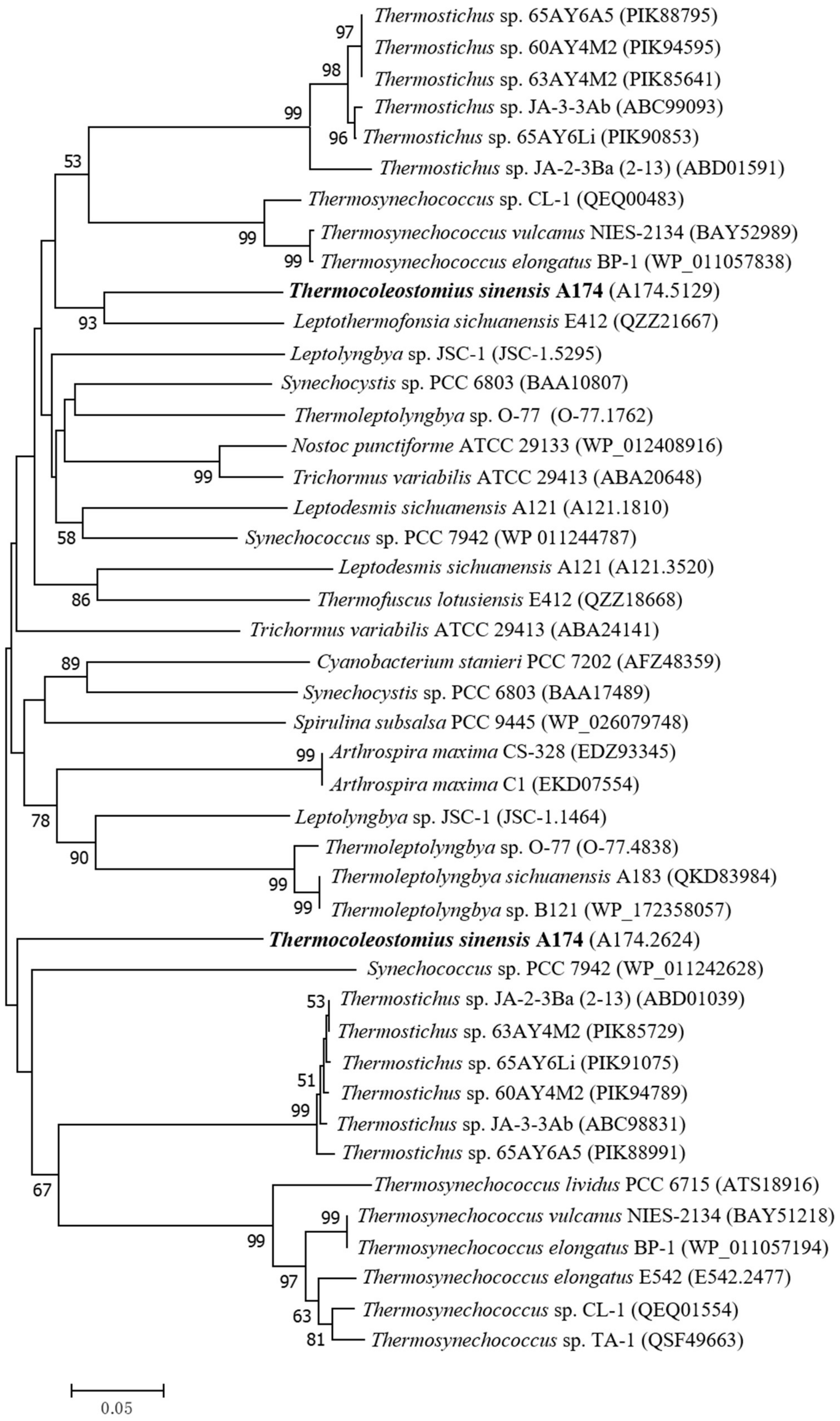

Supplementary Figure 9 Phylogenetic inference of variants of putative *cmpD* gene protein sequences. The accession numbers underscored refers to the gene IDs in Supplementary Table 1 of this study and Supplementary Table 1 of the reference Tang et al. 2022 doi: 10.3389/fmicb.2022.876272.
Only bootstrap values > 50% are indicated at nodes. Scale bar = 1% substitutions per site.

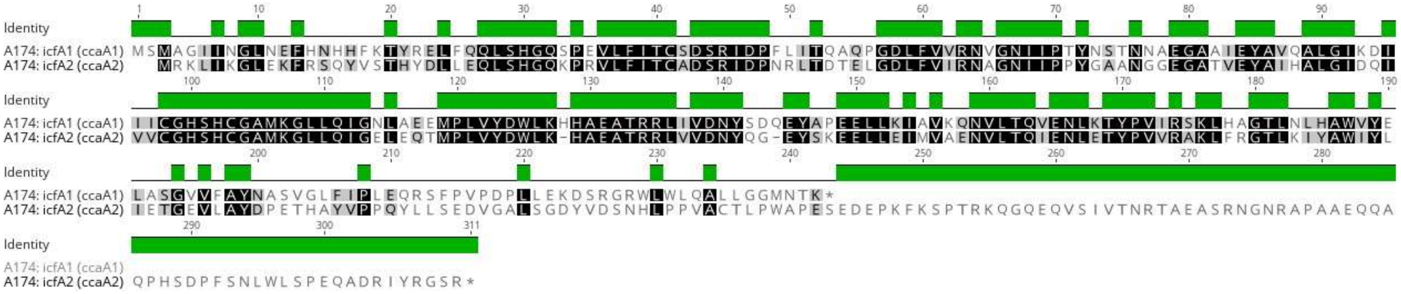

Supplementary Figure 10 Protein sequence alignment of two carbonic anhydrases ccaA1 and ccaA2 from *Thermocoleostomius sinensis* A174

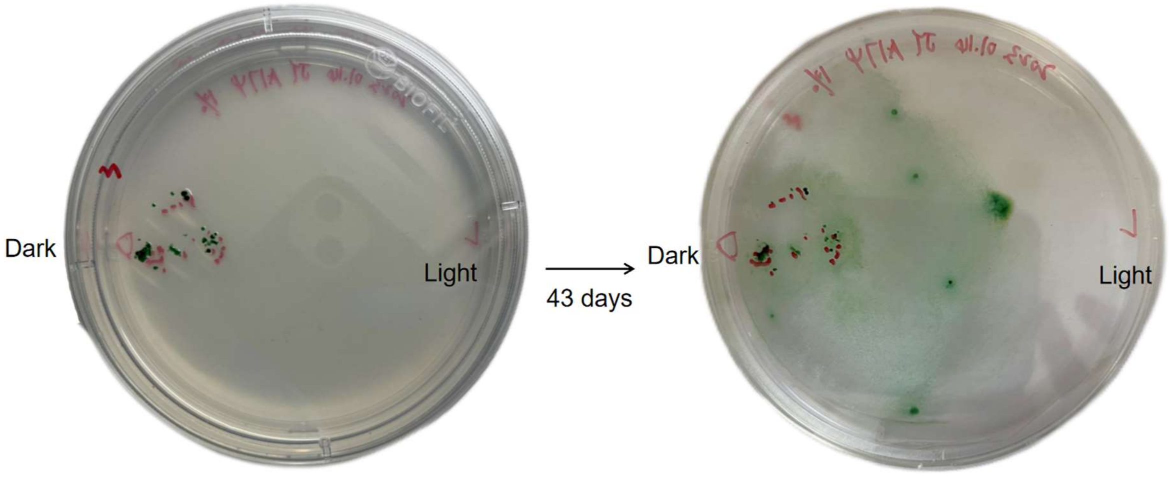

Supplementary Figure 11 Phototaxis capacity of *Thermocoleostomius sinensis* A174 under directional light

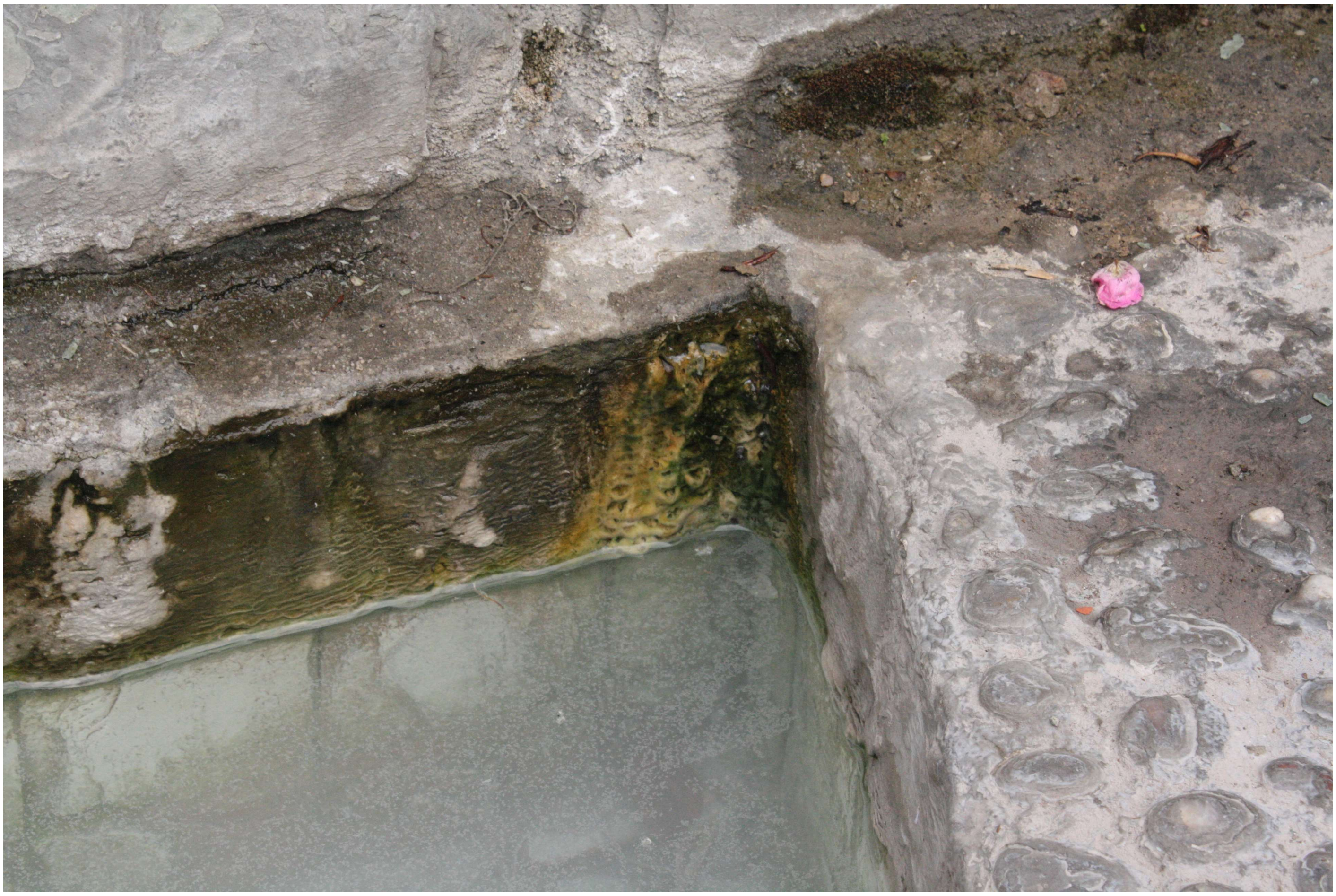

Supplementary Figure 12 Photography of the Erdaoqiao thermal spring in Ganzi Prefecture of Sichuan Province, China (30°05’14” N, 101°56’55” E) from where the *Thermocoleostomius sinensis* A174 strain was isolated.

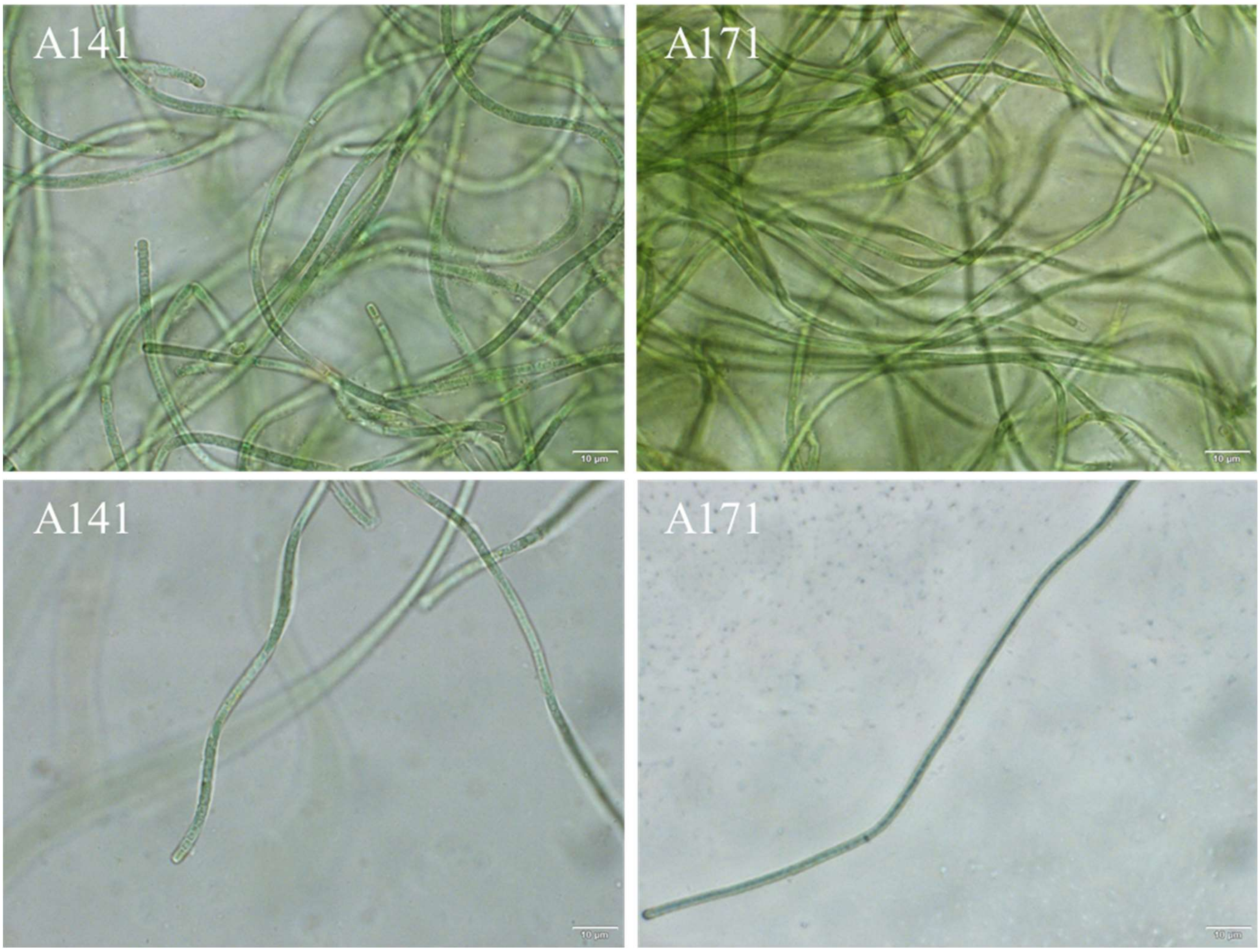

Supplementary Figure 13 Light micrographs of *Thermocoleostomius sinensis* sp. PKUAC-SCTA141 and A171. Scales = 10 µm.

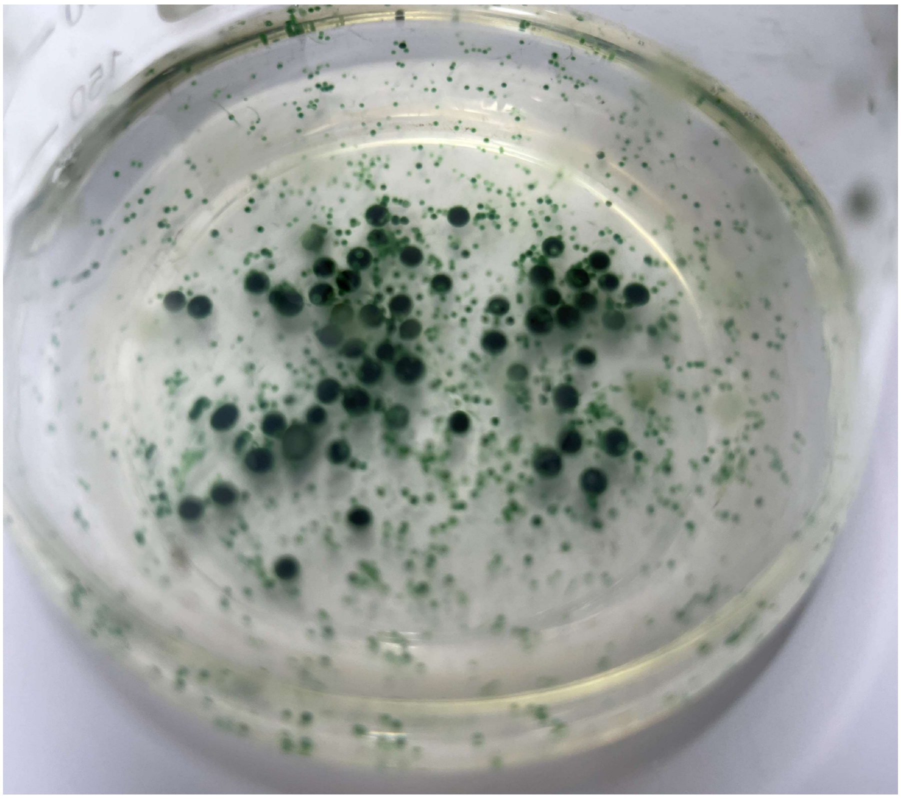

Supplementary Figure 14 The photography of trichoid pellets of *Thermocoleostomius sinensis* sp. PKUAC-SCTA174
